# Data-driven full-chain quality control for Xiaoer Chiqiao Qingre granules: from process risk management to intelligent product testing

**DOI:** 10.1186/s13020-026-01414-z

**Published:** 2026-05-12

**Authors:** Nan Wang, Yukun Chang, Yue Cheng, Jing Zhao, Chao Li, Lu Sun, Liang Feng, Yanjun Yang, Xiaobin Jia

**Affiliations:** 1 Jiangning Hospital of Chinese Medicine, School of Traditional Chinese Pharmacy, China Pharmaceutical University, Nanjing, 211198 People’s Republic of China; 2 Jiangsu Key Laboratory of Chinese Medicine and Characteristic Preparations for Paediatrics, Jumpcan Pharmaceutical Co., Ltd., Taixing, 225400 People’s Republic of China; 3 Nanjing Jiangning District Hospital of Chinese Medicine, Nanjing, 211100 People’s Republic of China

**Keywords:** Xiaoer Chiqiao Qingre granules, Quality by design, HPLC fingerprint, Physical fingerprinting, Design space for alcohol precipitation process, Machine vision

## Abstract

**Background:**

Xiaoer Chiqiao Qingre granules (XECQ) is a widely used traditional Chinese medicine for the treatment of pediatric wind-heat colds. However, its multi-step manufacturing process results in substantial inter-batch variability, posing a challenge for consistent quality control. Guided by the Quality by Design (QbD) concept, this study developed an integrated framework that combines process analysis, parameter optimization, and intelligent monitoring to improve batch-to-batch consistency and ensure medication safety.

**Methods:**

Within the QbD framework, HPLC-based multi-component quantification, chromatographic fingerprinting, and physical fingerprinting were employed to monitor quality across the entire manufacturing process, including extraction, concentration, alcohol precipitation, and decoction collection. Eleven active constituents were quantitatively characterized, and their relationships with key physicochemical properties were analyzed. The retention rates of terpenoids, flavonoids, and phenylethanoid glycosides were defined as critical quality attributes (CQAs). Critical process parameters were screened using a Plackett–Burman design, and the design space for the alcohol precipitation process was established through Box–Behnken response surface methodology. In addition, a machine vision system was developed for rapid, non-destructive evaluation of particle appearance.

**Results:**

Alcohol precipitation resulted in the greatest loss among the 11 monitored components, with an average reduction of 23.6%, whereas maackiain-7-O-glucoside exhibited a marked decrease of 38.1% during the concentration stage. After alcohol precipitation, the concentration factors of flavonoids, phenylethanoid glycosides, and terpenoids were 1.56, 1.53, and 1.50, respectively, suggesting a negative correlation between retention rate and compound polarity. Component redistribution during processing significantly reduced the relative standard deviation (RSD) of viscosity, indicating improved system homogeneity and a reversal of component–property correlations. Using these representative components as CQAs, robust regression models (Adj R^2^ > 0.85) were established to define a design space that maximized CQA retention while minimizing batch-to-batch variability. Furthermore, the machine vision system achieved 100% accuracy in both identification and abnormal batch rejection.

**Conclusion:**

This study proposes a holistic “Process Analysis-Parameter Optimization-Intelligent Monitoring” strategy, providing a robust framework for enhancing the quality control of XECQ and facilitating the transition of TCM manufacturing from empirically driven practices to data-driven processes.

**Graphical Abstract:**

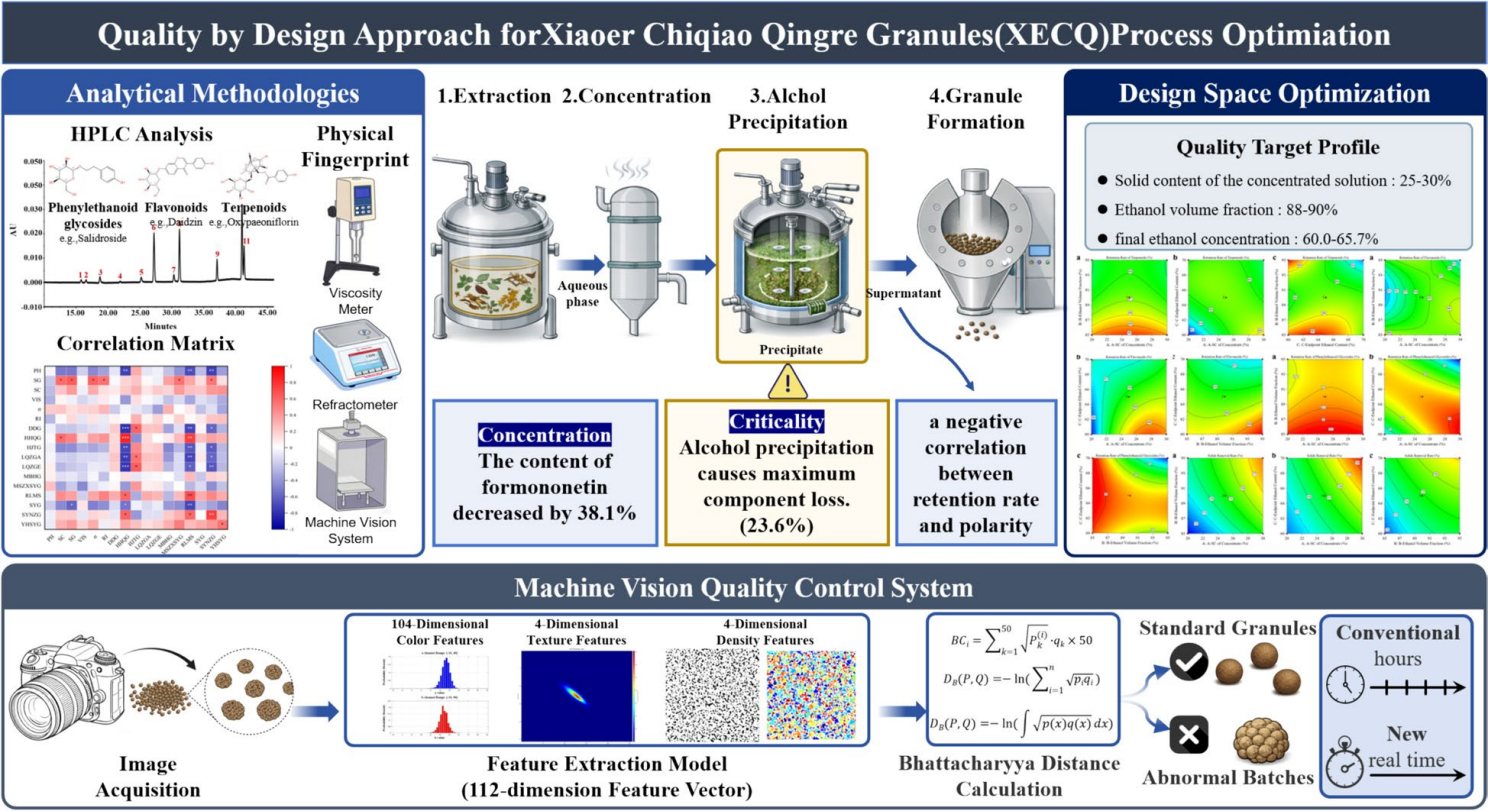

**Supplementary Information:**

The online version contains supplementary material available at 10.1186/s13020-026-01414-z.

## Introduction

The quality consistency of TCM compound formulations represents a core bottleneck that hinders their modernization and globalization. This challenge stems from the inherent complexity and variability of herbal materials, along with unavoidable fluctuations in the production process.

The traditional “quality by testing” (QbT) model relies on end-product inspection [1, 2], failing to proactively control quality fluctuations arising from critical sources of variation during production—such as process parameter drift and raw material variability. This results in an irreconcilable systemic disconnect between quality standards and clinical efficacy. Therefore, advancing the quality control paradigm from passive QbT to proactive QbD has become an industry consensus and an urgent necessity [3]. The QbD philosophy originated from the quality-by-design concept introduced by Joseph M. Juran's [4, 5] and was later formalized in the ICH Q8-Q10 guidelines. Its core involves systematically defining the quality target product profile (QTPP) to identify critical material attributes (CMAs), critical quality attributes (CQAs), and critical process parameters (CPPs), thereby establishing robust design spaces and control strategies [6–9]. This provides a scientific framework for integrating process analytical technology and enabling real-time release.

XECQ is composed of 14 TCM ingredients, including Forsythia suspensa (Thunb.) Vahl, Mentha canadensis L., Nepeta cataria L., Scutellaria baicalensis Georgi, and Glycyrrhiza uralensis Fisch. It is indicated for dispelling wind, relieving exterior syndrome, clearing heat, and resolving stagnation [10, 11].

Taking the commonly used pediatric medicine XECQ as an example, the challenges it faces in quality control are particularly severe and representative. XECQ are composed of 14 Chinese medicinal herbs, including *Glycine max (L.)* Merr. (9.0%), *Forsythia suspensa* (Thunb.) Vahl (12.0%), *Mentha canadensis* L. (6.0%), *Glycyrrhiza uralensis* Fisch. (5.0%), *Rheum palmatum* L. (5.0%), *Nepeta cataria* L. (6.0%), *Gardenia jasminoides* J.Ellis (parched, 5.0%), *Artemisia caruifolia* Buch.-Ham. ex Roxb. (9.0%), *Bupleurum chinense* DC. (6.0%), *Areca catechu* L. (4.0%), *Magnolia officinalis* Rehd.et Wils. (9.0%), *Scutellaria baicalensis* Georgi (9.0%), *Pinellia ternata* (Thunb.) Ten. ex Breitenb. (9.0%), and *Paeoniae Radix* Rubra (6.0%) [10]. It possesses the effects of dispelling wind, relieving exterior syndrome, clearing heat, and resolving stagnation [11]. Clinically, it is primarily used to treat acute upper respiratory tract infections and hand, foot, and mouth disease in children [12, 13]. However, as a typical TCM compound preparation, despite the efficacy of XECQ has been proven, the complexity of its raw materials and production processes still poses industry-wide challenges for the product, including limited quality control indicators and poor batch-to-batch consistency. Given its status as a pediatric medication, there is an urgent need to establish a more robust quality control system. Children’s organs are still developing, and their absorption, distribution, metabolism, and excretion (ADME) processes differ from those in adults, leading to unique pharmacodynamic characteristics. The synergistic effects of multiple components form the basis for the therapeutic efficacy of TCM formulas, but they may also increase the potential risk of pharmacokinetic interactions. This characteristic necessitates higher standards for formulation consistency in pediatric medications [14, 15]. Therefore, establishing a robust quality control system for XECQ based on the QbD philosophy holds significant practical and exemplary value.

Although the QbD concept provides a framework for systematic quality control, its application throughout the manufacturing process of complex TCM formulations still faces critical bottlenecks. The quality control system for Chinese patent medicines based on the QbD philosophy advocates systematic, risk-based strategies throughout the entire process [16–18] (Figure S1). This framework begins with defining the QTPP, which then guides the identification of CQAs [19, 20]. Through risk assessment, CQAs are linked to CPPs. Design of experiments (DoE) establishes design spaces to define optimal operating ranges, ultimately ensuring quality consistency via comprehensive control strategies [21–23]. This methodology collectively forms a closed-loop management system encompassing “objective definition-risk management-space optimization-strategy implementation”. However, most existing studies lack dynamic tracking and quantitative analysis of the synergistic evolution patterns between multiple components and key physical properties throughout the production process, leaving the “black box” issue unresolved. For instance, process optimization often relies on single or limited metrics without correlating with the overall retention efficiency of multiple active ingredient groups. This results in limited predictability and robustness of the design space. Meanwhile, end-of-line control predominantly depends on offline, destructive testing, making real-time monitoring and feedback adjustments during production challenging.

To address the aforementioned issues, this study selected terpenoids (paeoniflorin, oxypaeoniflorin, galloylpaeoniflorin, albiflorin), flavonoids (ononin, daidzein, genistein, wogonoside), and phenethyl glycosides (forsythoside A, forsythoside E, salidroside) as CQAs was based on a comprehensive consideration of pharmacological relevance, representativeness, process sensitivity, and QbD-oriented multi-component control logic [24]. Firstly, these compounds collectively represent the principal bioactive categories in XECQ—terpenoids, flavonoids, and phenylethanoid glycosides—which are widely recognized as the major anti-inflammatory and antiviral constituents of the constituent herbs [25]. Secondly, their documented mechanisms, including modulation of inflammatory signaling pathways, attenuation of oxidative stress, and protection of epithelial barrier function, are closely aligned with the pathological characteristics of pediatric wind-heat colds and acute upper respiratory tract infections, thereby linking their chemical stability to clinical efficacy [26]. Thirdly, these components exhibit diverse physicochemical properties (e.g., polarity, solubility, and stability) and are highly sensitive to critical manufacturing steps, particularly alcohol precipitation, enabling them to effectively reflect process-induced compositional redistribution and quality variation [11]. Finally, considering that the therapeutic effect of complex TCM formulations arises from multi-component synergy rather than a single dominant compound, defining CQAs at the level of representative component groups provides a more rational and holistic quality control strategy under the QbD framework. Together, these considerations justify the selection of the 11 compounds as scientifically and clinically relevant CQAs for XECQ.

To systematically address the aforementioned challenges, this study goes beyond the conventional application of QbD by uncovering mechanistic insights and establishing XECQ-specific control strategies. Unlike previous studies that primarily correlate chemical markers with process parameters at isolated stages, we performed dynamic, multidimensional tracking of both chemical components and physicochemical properties across the entire manufacturing chain. Notably, we identified a reversal phenomenon in component–physical property correlations during the alcohol precipitation stage, revealing that the restructuring of the material system fundamentally alters the interaction pattern between multi-component composition and macroscopic physical attributes. This finding provides new mechanistic evidence for resolving the long-standing “black box” issue in TCM process evolution. Recognizing alcohol precipitation as the major quality-loss node for XECQ, we further constructed a data-driven design space specifically tailored to this critical step, centered on the synergistic retention of terpenoids, flavonoids, and phenylethanoid glycosides. Rather than optimizing single indicators, the established model integrates multi-group retention efficiency and inter-batch robustness, thereby transforming empirical parameter adjustment into a statistically validated and mechanistically interpretable control strategy. In addition, to extend quality assurance from process optimization to intelligent product verification, we developed a 112-dimensional feature-fusion machine vision framework that integrates color, texture, and density descriptors with dynamic safety interval normalization and Bhattacharyya similarity metrics. This high-dimensional feature architecture enables real-time, non-destructive discrimination of compliant and abnormal batches, forming a data-coupled extension of the QbD system. Collectively, this study establishes a mechanistically interpretable and data-driven closed-loop architecture linking component evolution-physicochemical restructuring-design space construction-intelligent appearance verification.

This framework not only provides a dedicated quality control solution for XECQ but also demonstrates how deep process understanding and intelligent technologies can be structurally integrated to advance precision manufacturing of complex TCM formulations.

## Materials and methods

### Reagents and chemicals

Fifteen batches of intermediates from the production process of XECQ (namely, water extract, concentrated water extract, ethanol precipitation supernatant, and concentrated ethanol precipitation, corresponding to the extraction, concentration, ethanol precipitation, and re-concentration steps, respectively) were provided by Jichuan Pharmaceutical Group Co., Ltd. (Batch Nos.: 23110064A-23110204A), all meeting GMP requirements. XECQ ethanol-precipitated concentrated extract (laboratory preparation, Batch No.: 20241124), XECQ reference granules (laboratory preparation, Batch Nos.: 202412011–202412015). The reference standard paeoniflorin (≥ 98.0%, Batch No.: HR22112B1) used in the experiment was purchased from Baoji Herbest Bio-Tech Co., Ltd. Oxypaeoniflorin (≥ 98.0%, Batch No.: PS010199), Galloylpaeoniflorin (≥ 98.0%, Batch No.: PS011182), Albiflorin (≥ 95.0%, Batch No.: PS011455), Ononin (≥ 98.0%, Batch No.: PS000671), Daidzin (≥ 98.0%, Batch No.: PS011911), Genistein (≥ 98.0%, Batch No.: PS000785), Wogonoside (≥ 98.0%, Batch No.: PS011071), Forsythoside A (≥ 98.0%, Batch No.: PS011810), Forsythoside E (≥ 95.0%, Batch No.: PS000591), and Salidroside (≥ 98.0%, Batch No.: PS011472) were all purchased from Chengdu Push Bio-technology Co., Ltd. Dextrin (Batch No.: 240104042D) was purchased from Nanjing Chemical Reagent Co., Ltd. Chromatography-grade phosphoric acid (Batch No.: B2211238) was purchased from Shanghai Aladdin Biochemical Technology Co., Ltd. Chromatography-grade methanol (Batch No.: 24015464) was purchased from Tedia Company, Inc.. Experimental water was prepared using a Milli-Q ultra-pure water system (Merck Millipore, USA).

### HPLC assay and fingerprint analysis method

#### Preparation of standard solution

Precisely weigh each reference substance, dissolve in methanol, and dilute to 5 mL to prepare a solution containing 256.84 μg/mL oxypaeoniflorin (YHSYG), paeoniflorin (SYG) 1558.54 μg/mL, galloylpaeoniflorin (MSZXSYG) 217.02 μg/mL, albiflorin (SYNZG) 584.92 μg/mL, ononin (MBHG) 219.54 μg/mL, daidzin (DDG) 596.48 μg/mL, genistein (RLMS) 208.74 μg/mL, wogonoside (HHQG) 395.61 μg/mL, forsythoside A (LQZGA) 1188.10 μg/mL, forsythoside E (LQZGE) 225.15 μg/mL, and salidroside (HJTG) 234.43 μg/mL. Working curve solutions were prepared by methanol gradient dilution, filtered through a 0.22 µm membrane filter, and analyzed by HPLC (Figure S2).

#### Preparation of test solution

The aqueous extract was diluted with ultrapure water at a ratio of 1:2, while the concentrated aqueous extract was diluted at 1:20. Similarly, the ethanol precipitation supernatant was diluted with 65% ethanol at 1:2, and the concentrated ethanol precipitation fraction was diluted at 1:20. The diluted solutions were centrifuged at 13,000 rpm for 10 min, and the supernatants were collected. After filtration through a 0.22 µm membrane filter, the filtrates were subjected to HPLC analysis (Figure S3).

#### Chromatographic conditions

Chromatographic analysis was performed using a Water Acquity HPLC C18 column (4.6 mm × 250 mm, 5 μm). The mobile phase consisted of methanol (A) and 0.1% phosphoric acid aqueous solution (B). The gradient elution program is set as follows: 0–30 min, B decreases linearly from 90 to 55% (A increases from 10 to 45%); 31–35 min, B decreases from 55 to 39% (A increases from 45 to 61%); 36–50 min, B decreases from 39 to 35% (A increases from 61 to 65%); 51–55 min, B returns to 90% (A decreases to 10%) for column re-equilibration. The flow rate was 0.8 mL/min, column temperature was 35 ℃, detection wavelength was 278 nm, and injection volume was 10 μL. This method was employed for determining the content of marker components and constructing HPLC fingerprint profiles.

#### Methodological validation

In accordance with the general rules of volume IV of the 2020 edition of the Chinese pharmacopoeia, validate the specificity, linearity, precision, repeatability, stability, and recovery rate of the assay method. Simultaneously, evaluate the precision, repeatability, and stability of the HPLC fingerprint profile.

In accordance with the general requirements of Volume IV of the Chinese Pharmacopoeia (2020 edition), the specificity, linearity, precision, repeatability, stability, and recovery of the assay method were validated. Meanwhile, the precision, repeatability, and stability of the HPLC fingerprint were evaluated.

#### HPLC fingerprint similarity analysis

Based on the chromatographic data obtained from all batches of samples by HPLC analysis, reference spectra 1-124A, 2-124A, 3-124A, and 4-124A were selected using the Chinese Herbal Medicine Chromatographic Fingerprint Similarity Evaluation System Software (2012 Edition). A reference spectrum (R) was then generated by the average method with a time window of 0.1 min. Full-spectrum peak matching was performed, and the similarity was calculated.

### Establishment and analysis method of physical property fingerprint spectrum for XECQ intermediates

#### Determination of physical properties of XECQ intermediates

All test samples were equilibrated at 25 ℃, followed by thorough dissolution and homogenization via ultrasonication for 10 min. Following the general rules of the 2020 edition of the Chinese pharmacopoeia, the following parameters were simultaneously measured: specific gravity (SG), solid content (SC), pH, viscosity (VIS), conductivity (*σ*), and refractive index (RI). Each parameter was measured in triplicate, with results averaged. The obtained data were used for constructing the physical fingerprint.

#### Construction and analysis of physical property fingerprint profiles

Based on pharmacopoeia regulations and literature research, the numerical ranges for each physical property were determined, and the measurement indicators were standardized to values within the range of 0–10. The value ranges for each physical property indicator and the corresponding standardization conversion formulas are presented in Table 1.

**Table 1:** Standardized conversion method for physical properties of intermediate materials in key process steps of XECQ

Physical properties parameters	Parameter abbreviations	Unit	Numerical range	Conversion formula
Relative density	SG	–	0 ~ 2	5x
pH	pH	–	0 ~ 7	10x/7
Solid content	SC	%	0 ~ 50	x/5
Viscosity	VIS	mPa·s	0 ~ 120	x/12
Electrical conductivity	*σ*	mS/cm	0 ~ 20	x/2
Refractive index	RI	–	1 ~ 1.5	20 (x−1)

When the standardized values of six physical indicators, including SG and pH are all set to 10, connecting these points forms a regular hexagon. In contrast, connecting the standardized conversion values obtained from actual measurements of these physical indicators forms an irregular hexagon, which represents the physical property fingerprint spectrum of the intermediate in the critical process step of XECQ. Based on the physical property fingerprint spectrum of key intermediates in the XECQ process, the physical parameters of products from 15 batches across various production stages were analyzed. The physical property fingerprint spectra of XECQ intermediates were constructed using Origin software. These spectra were evaluated using both intuitive assessment and similarity evaluation methods. Correlations for each physical parameter were calculated both within and across stages to assess batch-to-batch and step-to-step quality consistency and variability.

### Study on the quantity and proportional relationships of 11 indicator components across key process stages of XECQ

By integrating the contents of SC, SG, and each indicator component, the proportion of 11 indicator components and their respective groups within the total solid content was calculated. This analysis examined the quantitative and proportional relationships among three component categories and 11 representative constituents across four key process stages of XECQ. The calculation formula is as follows:

1 $$Percentage\,by\,weight \left( \% \right) = \frac{Component\,concentration}{{Solid\,content\,\times\,Relative\,density\,\times\,Water\,density}}$$

Based on the quantitative results of multiple indicator components and the measured physical property parameters, the proportional ratios of the 11 indicator components within the total solid content were calculated for different batches of XECQ across various process stages. The resulting data were subsequently subjected to correlation analysis and multivariate statistical analysis combined with the physical property parameters.

### Optimization of the alcohol precipitation process and design space establishment for XECQ

Evaluation metrics for the alcohol precipitation process (active ingredient retention rate, solid removal rate) and analysis of potential process risks are presented in Supplementary Material S1.

#### Preparation of XECQ water extract concentrate and alcohol precipitation steps

The water extract concentrate of XECQ was pooled and concentrated from multiple batches to yield a concentrate with a total solid content of 37.3% (dried at 105 ℃ to constant weight), which served as the raw material for ethanol precipitation.

A 10 g portion of the concentrate raw material was precisely weighed, and the solid content was adjusted to the experimental requirement using a specific volume of deionized water. After thorough mixing, an ethanol solution of the corresponding volume fraction was added at the flow rate specified in the Plackett–Burman design scheme, accompanied by mechanical stirring at the prescribed speed. The addition of ethanol was stopped upon reaching the set volume. The mixture was then allowed to stand for static settling at the designated temperature. After a specified period, the mixture was filtered, and the precipitate was dried in an oven at 50 ℃ to constant weight and then weighed. The supernatant was collected, and its volume was measured for subsequent testing.

#### Plackett–Burman experiments for screening key process parameters

Using Design Expert 11.0 software, the following CQAs were selected: terpenoid retention rate (Y_1_), flavonoid retention rate (Y_2_), phenethyl glycoside retention rate (Y_3_), and solid removal rate (Y_4_). The screening factors included: solid content in the concentrate (A), ethanol volume fraction (B), ethanol concentration at the endpoint of ethanol precipitation (C), ethanol addition flow rate (D), stirring speed (E), static temperature (F), and static time (G). The levels of each factor are detailed in Table 2.

**Table 2:** Plackett–Burman design factor levels

Factor	Low level (−1)	High level (1)
A: Solid content of concentrate (%)	20	30
B: Volume fraction of ethanol (%)	85	95
C: Alcohol content at the endpoint of alcohol precipitation (%)	60	70
D: Alcohol addition flow rate (mL/min)	20	60
E: Stirring speed (r/min)	320	640
F: Standing temperature (℃)	4	25
G: Settling time (h)	12	24

#### Box-Behnken response surface design for optimizing alcohol precipitation process

Using Design Expert 11.0 software, a three-factor, three-level Box-Behnken design was established with the retention rates of Y_1_ terpenoids, Y_2_ flavonoids, and Y_3_ phenethyl glycosides, along with the solid removal rate (Y_4_), as CQAs. This design investigated the quantitative relationships among the three CQAs and the concentration variables: solid content in the concentrate (A), ethanol volume fraction (B), and alcohol content at the end of ethanol precipitation (C). The alcohol addiction rate was fixed at 20 mL/min, stirring speed at 320 r/min, incubation temperature at 4 ℃, and incubation time at 24 h. After alcohol addition, stirring continued for 5 min for screening. The Box-Behnken design plan is shown in Table 3.

**Table 3:** Box-Behnken design factor levels

Factor	Low level (−1)	Medium level (0)	High level (1)
A: Solid content of concentrate (%)	20	25	30
B: Volume fraction of ethanol (%)	85	90	95
C: Final alcohol content after precipitation (%)	60	65	70

#### Response surface modeling

The retention rates of terpenoid components (Y_1_), flavonoid components (Y_2_), phenethyl alcohol glycoside components (Y_3_), and solid removal rate (Y_4_) are calculated as follows:

2 $$Y_{1} = \frac{Terpenoid\,content\,in\,alcohol\,precipitation\,supernatant}{{Terpenoid\,content\,in\,concentrate}} \times 100\%$$

3 $$Y_{2} = \frac{Flavonoid\,content\,in\,alcohol\,precipitation\,supernatant}{{Flavonoid\,content\,in\,concentrate}} \times 100\%$$

4 $$Y_{3} = \frac{Phenylethanol\,glycoside\,content\,in\,alcohol\,precipitation\,supernatant }{{Phenylethanol\,glycoside\,content\,in\,concentrate}} \times 100\%$$

5 $$Y_{4} = \frac{Total\,mass\,of\,solids\,precipitated\,after\,alcohol\,precipitation}{{Total\,solids\,in\,concentrate}}$$

Model fitting was performed using ordinary linear regression and stepwise regression to examine the influence of process parameters on corresponding values:

6 $$Y\, = \,a_{0} \, + \,\sum {a_{i} x_{i} }$$

Here, Y represents the retention rate of active ingredients or solid removal rate, where a_0_ denotes the constant term, a_i_ denotes the regression coefficient, and x_i_ denotes the respective process parameters. By comparing the model performance under different conditions (such as Adj *R*^*2*^, predicted *R*^*2*^, and AIC/BIC values), the optimal simplified model is selected to achieve the screening of CPPs.

General linear regression combined with stepwise regression was performed to examine the influence of the process parameters on their respective response values (Eq. 6). This linear regression analysis served primarily to assess the independent effects of each parameter, enabling preliminary screening. For the final mathematical model aimed at process space optimization and prediction, a simplified cubic model including linear, interaction, and quadratic terms was adopted as the foundational form. Stepwise regression was then applied to fit the CQAs (Y1, Y2, Y3, Y4) and their corresponding CPPs (A, B, C).

#### Model validation and diagnostics

Based on the measured active ingredient contents, the retention rate of each component and the solid removal rate were calculated by substituting the values into Eq. (5). A cubic polynomial response surface model was established using Design‑Expert 11.0 software. Stepwise regression was performed to screen significant factors, with model optimization conducted using adjusted R^2^, predicted R^2^, and AIC/BIC as indicators. Additionally, 200 permutation tests were carried out to evaluate potential overfitting of the model.

### Machine vision-based method for evaluating particle quality consistency

#### Image acquisition system setup

**Fig. 1:**
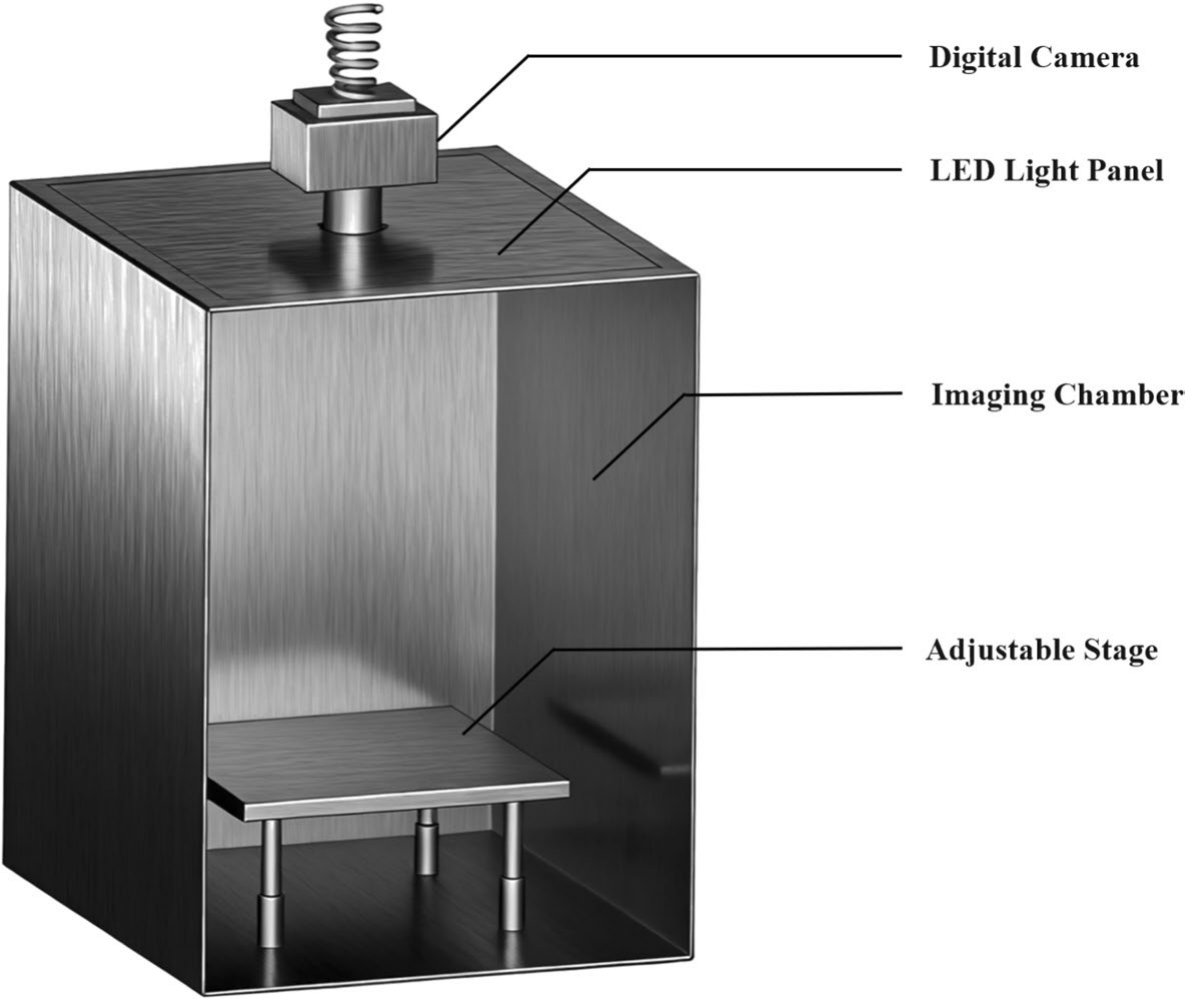
Particle digital image acquisition system

The machine vision system for particle image acquisition consists of an image acquisition unit and a processing unit. The acquisition unit comprises a digital camera, an LED light panel, a sealed chamber, and a height-adjustable specimen stage to ensure consistency and reproducibility of the imaging environment (Fig. 1). The processing unit is managed by a high-performance computer equipped with MATLAB 2021b (MathWorks Inc., USA), which performs all image processing and quality assessment algorithms.

#### Image processing and quality assessment workflow

The system workflow comprises three primary modules: Data layer, processing layer, and output layer. Standard particle images (G1–G11) serve as the training set for establishing quality benchmarks. Independent standard particles (G12–G14) and reference particles (RG1–RG5) function as the test set for system performance validation. All images are captured under strictly uniform lighting, resolution, and format (JPG) conditions. The processing layer performs image preprocessing and feature analysis. During preprocessing, the system automatically extracts five standardized subregions (400 × 400 pixels) from each raw image for subsequent analysis. In the core processing stage, a multidimensional analysis approach integrating color (LAB space), texture, and morphological features is employed to extract high-dimensional feature vectors from each sample for intelligent evaluation (detailed feature definitions and extraction algorithms are provided in Supplementary Material S2). The output layer ultimately outputs the quantified features of each sample along with its quality assessment result, classified into three grades: “Accept,” “Review,” or “Reject.”

### Statistical analysis

As mentioned earlier, the similarity of HPLC fingerprint spectra from different batches was analyzed using the Chinese Medicine Chromatographic Fingerprint Similarity Evaluation System Software (2012 Edition). Design Expert 11.0 software was used to optimize the XECQ ethanol precipitation process and establish its design space. For quality assessment of the finished XECQ product, a self-developed granular machine vision quality evaluation system was applied. Additionally, Pearson correlation analysis was performed with SPSS 22.0, PCA dimensionality reduction was conducted with Origin 2023, and an OPLS-DA model was established in SIMCA 14.1. After model robustness was confirmed through 200 permutation tests (*P* < 0.05), key differential variables were extracted using a VIP > 1 threshold.

## Results

### HPLC method validation and XECQ intermediate quality analysis

#### Method validation

A systematic validation of the HPLC method for analyzing XECQ process intermediates was conducted. Specificity tests showed that all 11 target components exhibited corresponding chromatographic peaks in the water extract, concentrated water extract, ethanol precipitation supernatant, and concentrated ethanol precipitate. No interference was observed in blank solvents (ultrapure water, 65% ethanol, methanol). After 2- to 64-fold gradient dilution of mixed standard solutions (HB_0_), all analytes exhibited excellent linearity (linear regression coefficient *r* ≥ 0.999) (Table S1). The method demonstrated good repeatability (*n* = 6), instrument precision (*n* = 6), and 24 h solution stability (*n* = 7), with peak area relative standard deviation (RSD) values ≤ 3.00% (Table S2). In the spiked recovery test, the average recovery rates for 11 components ranged from 95.10 to 103.37%, with RSD values ≤ 3.64%. Take the mixed standard with the lowest concentration from the above, dilute it with methanol, inject 10 μL, and calculate the limit of detection (LOD) based on a signal-to-noise ratio (S/N) of approximately 3. Calculate the limit of quantification (LOQ) based on a S/N of approximately 10. The detailed results for the LOD and LOQ for the 11 components, HJTG (LOD: 0.6456 μg/mL, LOQ: 2.2098 μg/mL), LQZGE (LOD: 0.6541 μg/mL, LOQ: 1.9146 μg/mL), YHSYG (LOD: 0.6742 μg/mL, LOQ: 0.9302 μg/mL), SYNZG (LOD: 0.7048 μg/mL, LOQ: 11.6619 μg/mL), SYG (LOD: 0.8747 μg/mL, LOQ: 8.2795 μg/mL), DDG (LOD: 0.6242 μg/mL, LOQ: 0.3338 μg/mL), MSZXSYG (LOD: 0.6073 μg/mL, LOQ: 0.6760 μg/mL), LQZGA (LOD: 0.4887 μg/mL, LOQ: 0.5208 μg/mL), MBHG (LOD: 0.5226 μg/mL, LOQ: 0.3223 μg/mL), HHQG (LOD: 0.0301 μg/mL, LOQ: 0.1400 μg/mL), and RLMS (LOD: 0.0555 μg/mL, LOQ: 0.1947 μg/mL) are presented in Table S3. Results indicate that this method exhibits strong specificity, excellent linearity, high precision and accuracy (Table S4), and satisfactory solution stability. It is suitable for the quantitative analysis of target components in XECQ intermediates and finished products.

#### Determination of indicator component content

Content determination was performed on test solutions comprising 15 batches of XECQ water extracts (Table S5), water extract concentrates (Table S6), alcohol precipitation supernatants (Table S7), and alcohol precipitation concentrates (Table S8). The calculated component content of each batch of samples provides a basis for subsequent research.

### HPLC fingerprinting method validation and similarity assessment

#### Method validation

The method validation included assessments of repeatability, precision, and 24 h stability, with all measurements performed using forsythoside A as the internal reference peak. Six independent samples were prepared from the ethanol-precipitated concentrate of batch 23110064A for testing. The RSDs of relative retention time ranged from 0.01 to 0.09%, and those of relative peak area ranged from 0.29 to 1.94%, demonstrating good repeatability of the method.

The test solution (sample 4-064A) was injected six consecutive times under the chromatographic conditions. The RSD of relative retention time (RRT) and relative peak area (RPA) ranged from 0.01 to 0.04% and from 0.19 to 2.89%, respectively. Verification using the TCM Chromatographic Fingerprint Similarity Evaluation System (2012 Edition) showed that the similarity of all six injection profiles was 1.000, indicating excellent instrument precision. The same test solution was analyzed at 0, 2, 4, 8, 10, 12, and 24 h, yielding RSDs of 0.01–0.11% for RRT and 0.11–3.84% for RPA, demonstrating good solution stability within 24 h. These validation results confirm that the established method is reliable and suitable for subsequent analysis.

#### Establishment and similarity analysis of fingerprints

**Fig. 2:**
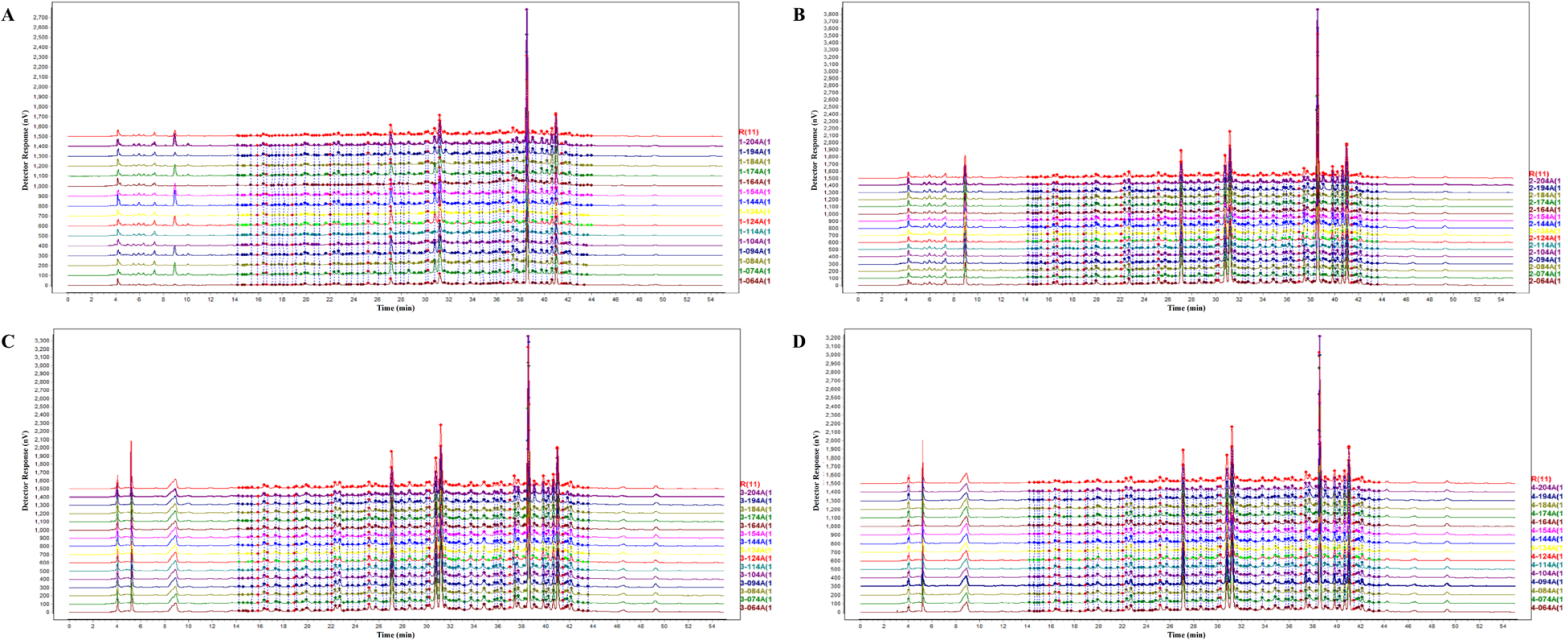
HPLC fingerprint profiles and their reference profiles. **A** 15 batches of aqueous extracts 1-064A ~ 1-204A, **B** 15 batches of concentrated aqueous extracts 2-064A ~ 2-204A, **C** 15 batches of ethanol precipitation supernatants 3-064A ~ 3-204A, **D** 15 batches of alcohol precipitation concentrates 4-064A to 4-204A

HPLC fingerprints and their corresponding reference chromatograms (Fig. 2) were established for 15 batches of samples at different processing stages (water extracts: 1-064A to 1-204A; water-extract concentrates: 2-064A to 2-204A; alcohol supernatant: 3-064A to 3-204A; alcohol precipitation concentrates: 4-064A to 4-204A). Similarity evaluation results (Table 4) showed that the RSD of the RRT for all common peaks was less than 1%, indicating highly consistent chromatographic separation behavior.

**Table 4:** Similarity evaluation results between 15 batches of water extracts, water extract concentrates, ethanol precipitation supernatants, ethanol precipitation concentrates, and their respective reference fingerprint spectra

Processing steps	Similarity														
	064A	074A	084A	094A	104A	114A	124A	134A	144A	154A	164A	174A	184A	194A	204A
Water extract	0.988	0.972	0.958	0.989	0.993	0.974	0.991	0.952	0.988	0.993	0.844	0.988	0.967	0.985	0.985
Water-soluble extract concentrate	0.992	0.998	0.990	0.994	0.999	0.998	0.998	0.996	0.998	0.999	0.998	0.997	0.992	0.982	0.990
Supernatant after ethanol precipitation	0.994	0.995	0.994	0.998	0.999	0.995	0.998	0.996	0.995	0.996	0.998	0.997	0.988	0.979	0.987
Concentrated alcohol precipitate	0.996	0.998	0.992	0.996	1.000	0.998	0.999	0.995	0.994	0.999	0.999	0.995	0.987	0.979	0.986

Similarity analysis further revealed that the water-extract concentrates, alcohol supernatant, and alcohol precipitation concentrates exhibited high inter-batch similarity, indicating good product consistency across these processing stages. Notably, three batches in the water extract stage (084A, 134A, and 164A) showed relatively low similarity (< 0.960) with their reference chromatograms, suggesting some variability in the starting materials. However, as the process advanced, intermediate products exhibited markedly improved similarity, indicating that the manufacturing process effectively homogenized material variations. Additionally, batches 184A, 194A, and 204A maintained acceptable similarity levels with their respective reference chromatograms in the water-extract concentrate, alcohol supernatant, and alcohol precipitation concentrate stages, although their similarity values were slightly below 0.990.

### Determination of physical properties and construction of physical property fingerprint profiles

#### Measurement of various physical properties

**Fig. 3:**
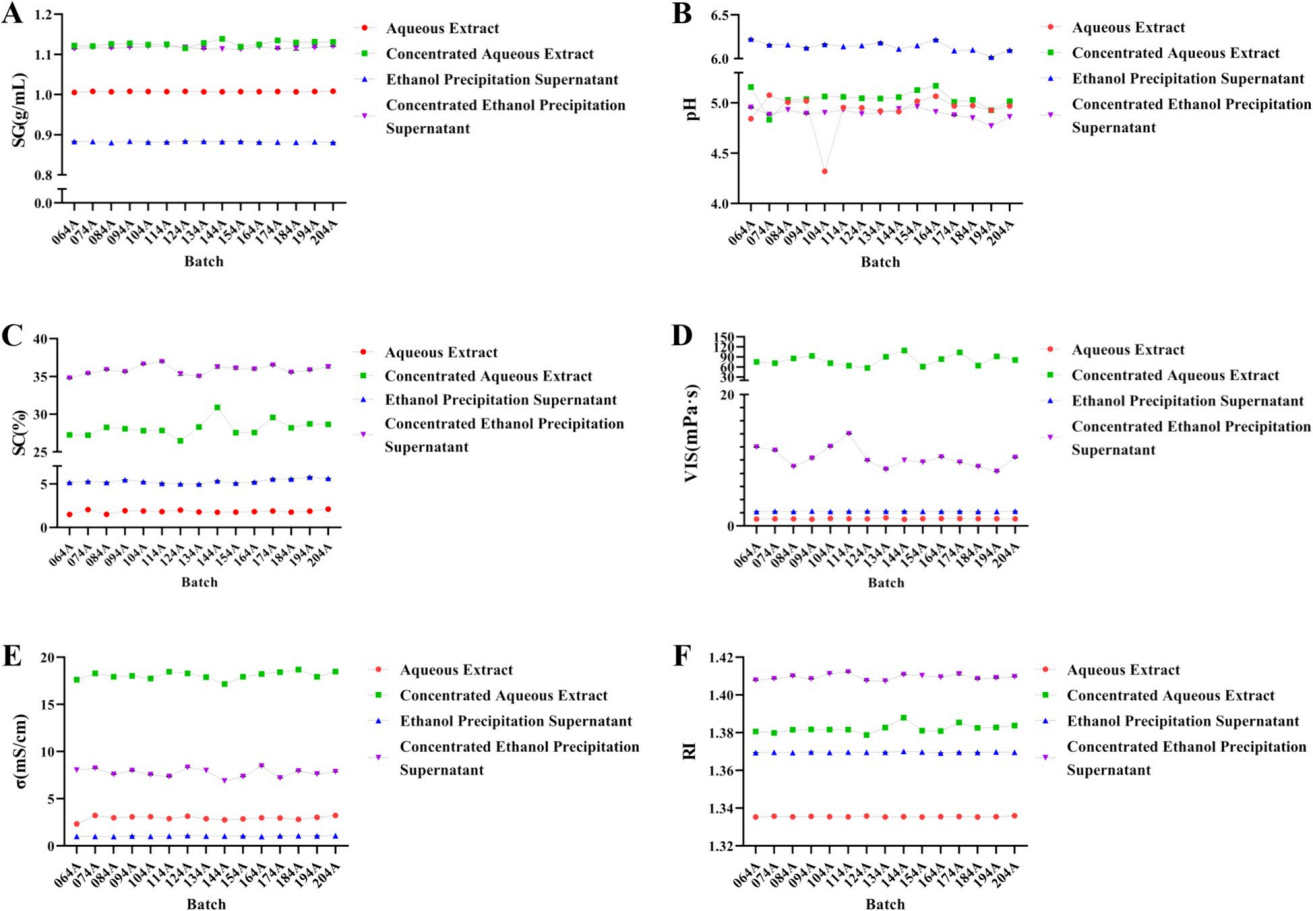
Characterization of physical parameters in key processing steps of XECQ. **A**–**F** SG, pH, SC, VIS, *σ*, and RI of water extracts, water-extract concentrates, alcohol supernatant, and alcohol precipitation concentrates from different XECQ batches

Six physical parameters (SC, SG, pH, *σ*, RI, and VIS) were measured for 15 batches of XECQ at four processing stages, including aqueous extract, aqueous extract concentrate, ethanol precipitation supernatant, and ethanol precipitation concentrate, as shown in Fig. 3A–F. Except for SG, which was determined in duplicate, all other physical parameters were measured in triplicate. The RSD values for parallel measurements at each stage were all below 3%, indicating good analytical precision and reliability. When evaluating inter-batch variability across the 15 batches at each processing stage, the overall RSD values of SG and *σ* were consistently below 1%, demonstrating high stability of these parameters throughout the process. In contrast, the overall RSD values of SC, RI, and VIS showed stage-dependent variations. Notably, the aqueous extract concentrate exhibited relatively greater fluctuations in VIS, *σ*, and SC compared with the other three stages, suggesting that the concentration step substantially alters the physicochemical state of the system. These changes likely reflect the progressive enrichment of solutes and modification of intermolecular interactions during solvent removal. However, despite these variations, the physical parameters of the ethanol precipitation supernatant and ethanol precipitation concentrate tended to reconverge, indicating a restructuring and stabilization of the system after alcohol precipitation.

#### Construction of physical fingerprint profiles

**Fig. 4:**
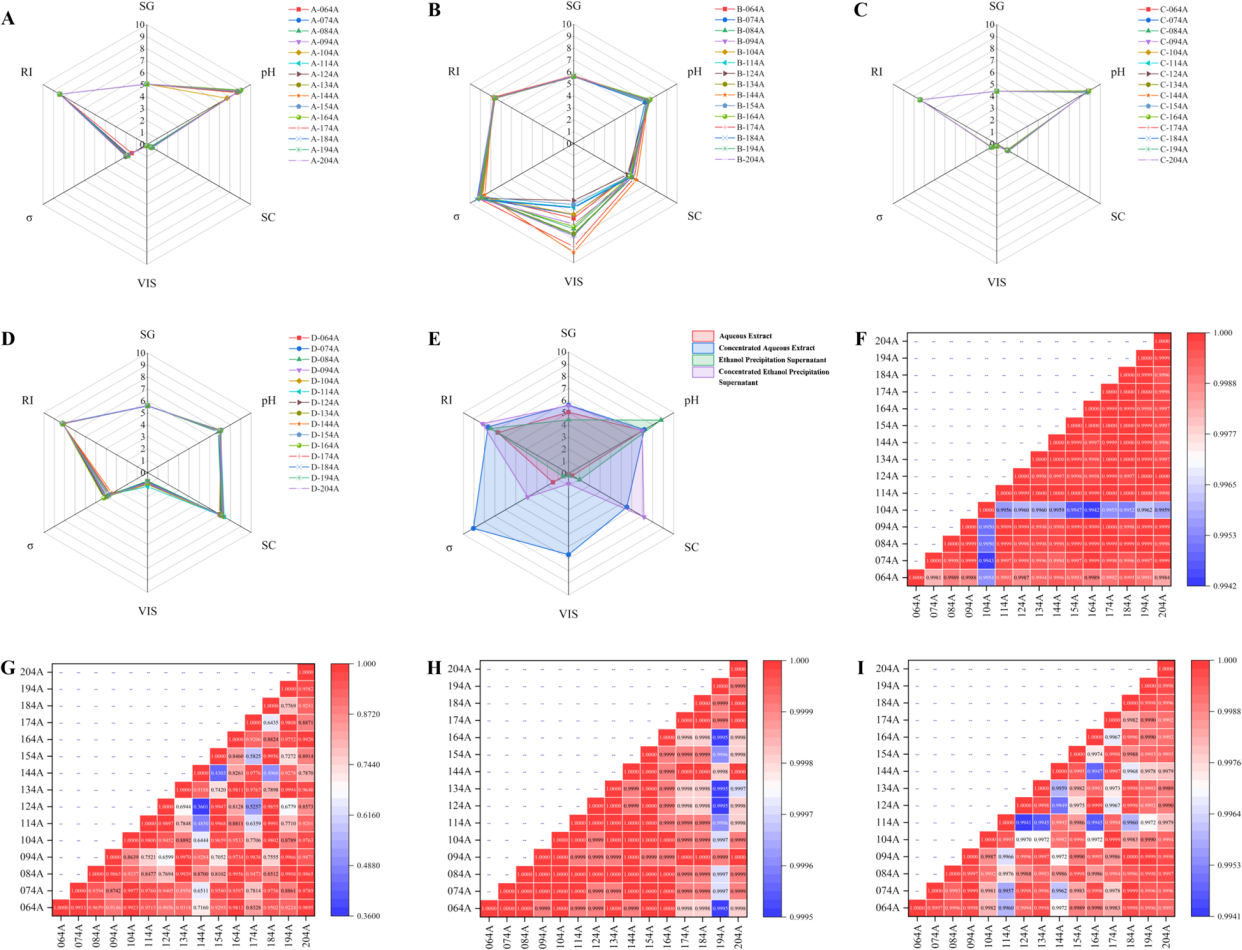
Development and evaluation of physical fingerprint profiles for key process steps of XECQ. **A**–**E** Physical fingerprint profiles of 15 batches of water extracts, water-extract concentrates, alcohol supernatant, and alcohol precipitation concentrates, with summary fingerprint profiles showing the average values of physical parameters at each step. **F**–**I** Similarity evaluation of 15 batches each for XECQ water extracts, water-extract concentrates, alcohol supernatant, and alcohol precipitation concentrates

The physical property fingerprint spectra of intermediates at key process stages of XECQ are shown in Fig. 4A–E. Based on these spectra, similarity analysis was conducted on 15 batches of intermediates to evaluate overall batch-to-batch consistency, with the results presented in Fig. 4F–I. During the four process stages, the pH of the samples exhibited an initial increase followed by a decrease, while the remaining physical parameters generally followed a pattern of initial increase, subsequent decrease, and then another increase. The overlay of the physical property fingerprint spectra reveals the similarities and differences in various quality indicators among intermediates from different stages and batches. The six physical property indicators for the aqueous extract, alcohol precipitation supernatant, and alcohol precipitation concentrate exhibited relatively concentrated distributions. In contrast, the aqueous extract concentrate showed fluctuations in three indicators, VIS, *σ*, and SC, with VIS exhibiting particularly pronounced variation. For VIS, batches 144A and 124A corresponded to the highest and lowest values, respectively.

Similarity analysis results indicate that in the water extract concentration stage, batches 114A, 124A, 144A, 154A, and 174A showed similarity scores below 0.800 with at least five other batches, suggesting that these batches deviated from the main group in overall properties. Specifically, the similarity between 144 and 114A, 124A, and 154A was below 0.500 and 0.700, respectively, while the similarity between 144 and 174A reached as high as 0.9776. These batches exhibited significant variability in relative density, solid content, viscosity, refractive index, and conductivity, indicating that 144A and 174A, as well as 114A, 124A, and 154A, may form a marginal group in terms of quality among the 15 batches.

### Correlation study on quality transfer in XECQ based on HPLC and physical fingerprints

#### Investigation of quantitative ratios among 11 indicator components across key processing steps of XECQ

**Fig. 5:**
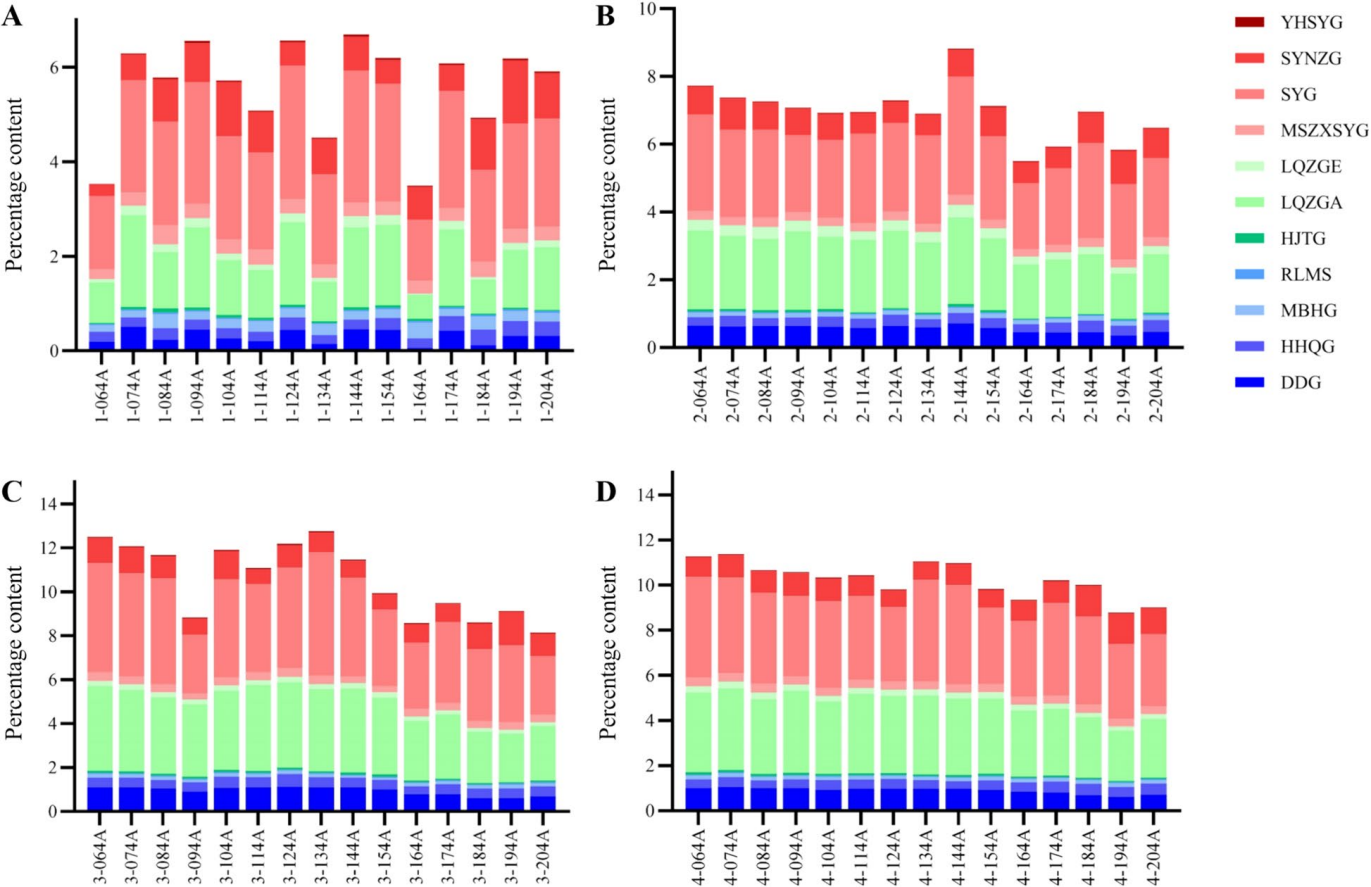
Quantitative ratios of indicator components per unit total solids in different processing steps of XECQ. **A** Ratios in 15 batches of water extracts. **B** Ratios in 15 batches of water-extract concentrates. **C** Ratios in 15 batches of alcohol supernatant. **D** Ratios in 15 batches of alcohol precipitation concentrates

Based on the quantitative results of multiple indicator components and the measured physical parameters, the quantitative ratios of the 11 indicator components per unit total solids were calculated across different processing steps for various XECQ batches (Fig. 5). As shown in Table 5, the percentages of the 11 indicator components and their subgroups per unit total solids in the water extracts, water-extract concentrates, alcohol supernatant, and alcohol precipitation concentrates were 5.57%, 6.95%, 10.56%, and 10.25%, respectively.

**Table 5:** Percentage of each component in the total solid content

Sample	DDG (%)	HHQG (%)	MBHG (%)	RLMS (%)	HJTG (%)	LQZGA (%)	LQZGE (%)	MSZSYG (%)	SYG (%)	SYNZG (%)	YHSYG (%)	Flavonoids (%)	Phenylethanol glycosides (%)	Terpenoids (%)	Total proportion (%)
Water extract	0.31	0.24	0.21	0.03	0.05	1.28	0.14	0.30	2.21	0.77	0.03	0.79	1.47	3.31	5.57
Water-soluble extract concentrate	0.56	0.29	0.13	0.03	0.05	2.02	0.28	0.26	2.53	0.77	0.03	1.01	2.36	3.58	6.95
Supernatant after ethanol precipitation	0.94	0.45	0.14	0.05	0.09	3.30	0.22	0.36	3.99	0.99	0.04	1.58	3.60	5.38	10.56
Concentrated alcohol precipitate	0.90	0.42	0.15	0.04	0.08	3.19	0.26	0.37	3.82	0.98	0.03	1.51	3.54	5.20	10.25

Regarding the structural composition per unit total solids, the proportional distribution among the three component categories shifted as the process advanced: from a ratio of flavonoids to phenylethanoid glycosides to terpenoids of 1.0: 1.9: 4.2 in the water extracts to 1.0: 2.3: 3.5 in the water extract concentrates, after which it stabilized. Specifically, from the water extract to the water extract concentrate stage, the proportion of terpenoids (YHSYG, SYNZG, SYG, and MSZXSYG) decreased, while that of phenylethanoid glycosides (LQZGE, LQZGA, and HJTG) increased. The proportion of flavonoids (RLMS, MBHG, HHQG, and DDG) showed a slight rise.

Throughout the entire process from water extract to alcohol precipitation concentrate, the proportions per unit total solids of all indicator components showed an increasing trend, except for MBHG and YHSYG, indicating effective impurity removal during alcohol precipitation. The decreased proportion of MBHG suggests significant loss of this component during purification, while the nearly unchanged proportion of YHSYG indicates that its loss rate was comparable to the impurity removal rate. The decline in MBHG and YHSYG proportions during the concentration step, together with the general reduction in most active components and total active components during the paste collection step, suggests that the concentration process may involve factors leading to the loss of active constituents.

During alcohol precipitation, the contents of flavonoids, phenylethanoid glycosides, and terpenoids per unit total solids increased to 1.56, 1.53, and 1.50 times their pre-precipitation levels, respectively. This indicates that the alcohol precipitation process effectively enhanced the purity of the indicator components, and the fold-changes were inversely correlated with the polarity of the three component classes.

#### Correlation analysis between indicator component contents and physical parameters

**Fig. 6:**
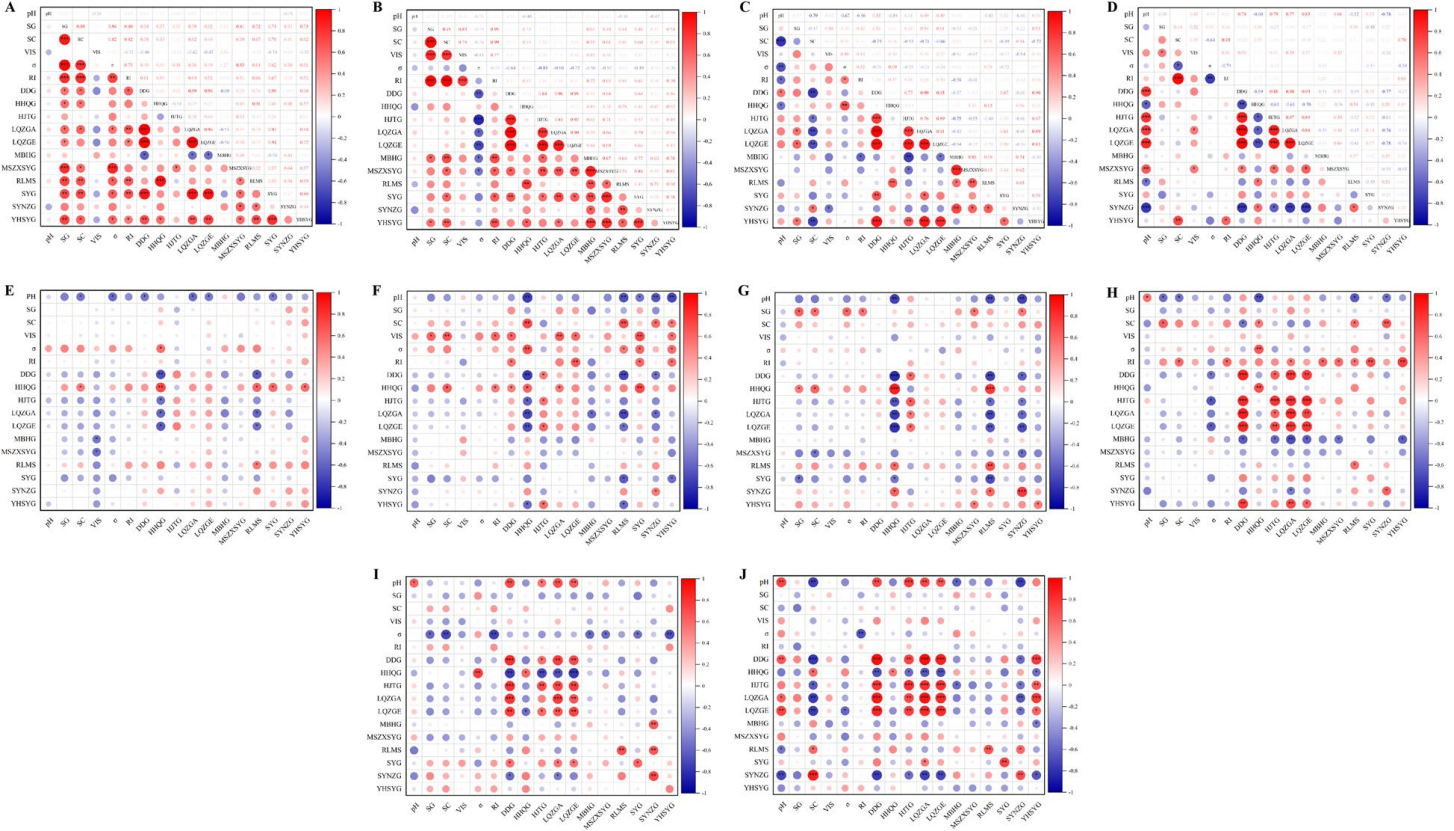
Correlation analysis of indicator component contents and physical parameters across processing steps. **A** Water extract-water extract correlation analysis. **B** Water-extract concentrate-water-extract concentrate correlation analysis. **C** Alcohol supernatant-alcohol supernatant correlation analysis. **D** Alcohol precipitation concentrate-alcohol precipitation concentrates correlation analysis. **E** Water extract-water-extract concentrate correlation analysis. **F** Water extract-alcohol supernatant correlation analysis. **G** Water extract-alcohol precipitation concentrates correlation analysis. **H** Water-extract concentrate-alcohol supernatant correlation analysis. **I** Water-extract concentrate-alcohol precipitation concentrate correlation analysis. **J** Alcohol supernatant-alcohol precipitation concentrates correlation analysis. ^*^P < 0.05, ^**^P < 0.01, ^***^P < 0.001

Bivariate correlation analysis was performed using SPSS 22.0 to investigate the relationships between physical parameters and the major component contents in 15 batches of water extracts, water-extract concentrates, alcohol supernatant, and alcohol precipitation concentrates (Fig. 6).

Following alcohol precipitation, a negative correlation was observed between four indicator components (Group 1: HJTG, LQZGA, LQZGE, and DDG) and three indicator components (Group 2: HHQG, RLMS, and SYNZG), while positive correlations were identified within each group. Additionally, Group 1 components showed a slight positive correlation with pH, whereas Group 2 components exhibited a slight negative correlation with this physical parameter. The intra-group positive correlations suggest that components within the same category share similar chemical properties and respond synchronously to identical processing conditions. The inter-group negative correlation indicates that the two component groups may display mutually exclusive behavior due to process selectivity, suggesting that relevant steps could be regulated to balance the yields of multiple components. The remaining components showed no significant correlations, although paeoniflorin generally aligned with Group 1, while ononin tended to associate with Group 2.

After the concentration step, strong positive inter-step transfer correlations were observed for eight components, including daidzein, indicating high transfer stability and minimal influence from unit operations (e.g., concentration, alcohol precipitation). In contrast, the negative or weak transfer correlations of MBHG, MSZXSYG, and YHSYG may reflect component transformation or loss.

#### Multivariate statistical analysis of indicator component content and physical property parameters

**Fig. 7:**
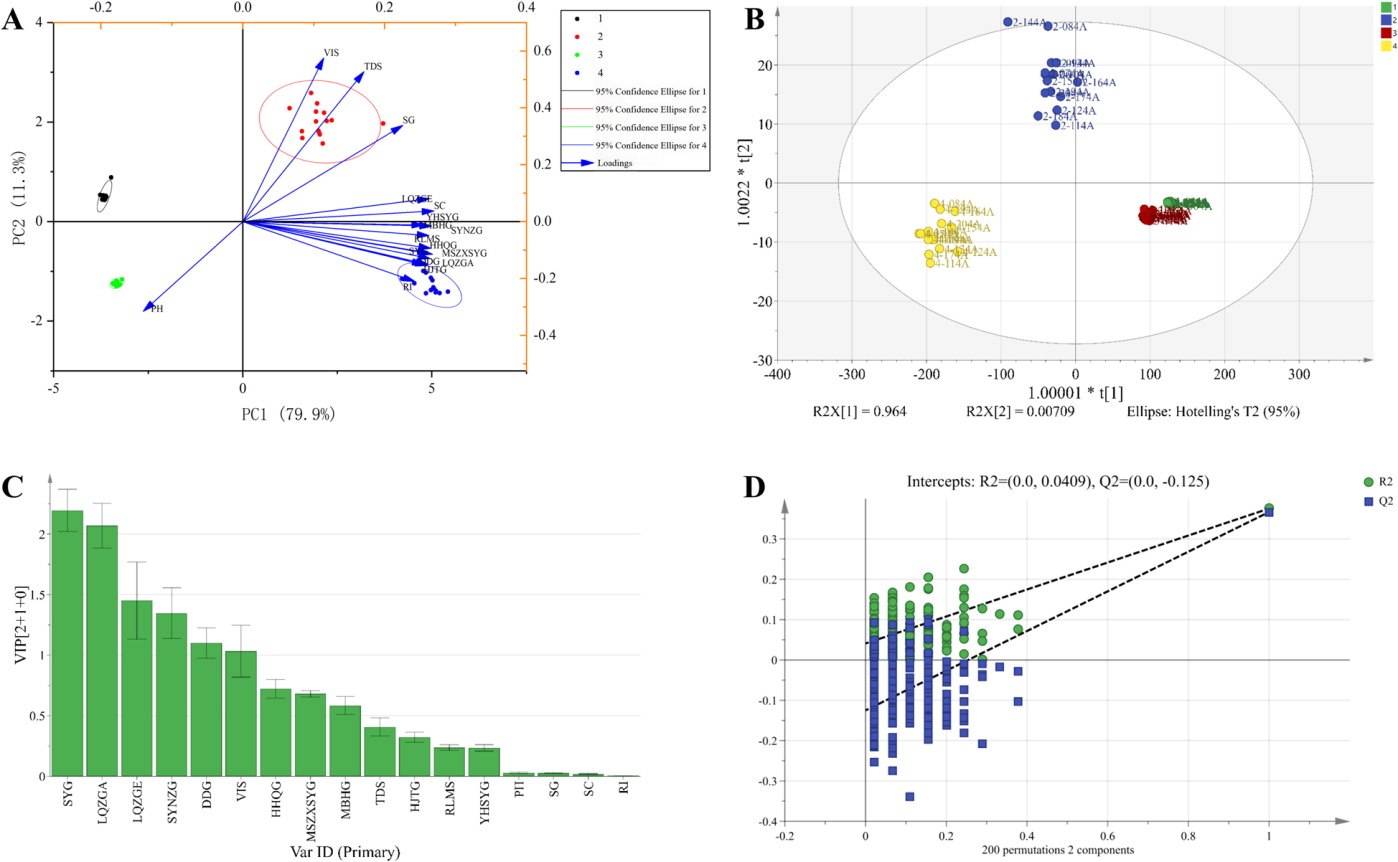
Multivariate statistical analysis of indicator component contents and physical parameters across processing steps. **A** PCA score plot of 15 batches of water extracts, water-extract concentrates, alcohol supernatant, and alcohol precipitation concentrates. **B** OPLS-DA score plot of the same sample sets. **C** OPLS-DA VIP values of the analyzed samples. **D** Permutation test plot for OPLS-DA validation. In the figure, Number 1 corresponds to the aqueous extract sample, Number 2 corresponds to the concentrated aqueous extract sample, Number 3 corresponds to the alcohol-precipitated supernatant sample, and Number 4 corresponds to the concentrated alcohol-precipitated supernatant sample

Principal component analysis extracted three principal components from 17 variables, accounting for 97.63% of the cumulative variance, indicating that the model effectively captured most of the data variation. pH, SG, and SC were identified as the primary contributors to batch-to-batch variation in process intermediates across different stages. Hotelling’s *T*^2^ test results showed that samples from all stages fell within the 95% confidence limits. The PCA score plot (Fig. 7A) further demonstrated that the intermediates from 15 batches exhibited clear intra-class clustering and inter-process separation across the four manufacturing stages.

The OPLS-DA model analysis results indicate that the independent variable fit index (*R*^*2*^_*x*_) is 0.992, the dependent variable fit index (*R*^2^_y_) is 0.64, and the model predictive capability index (*Q*^*2*^) is 0.63. Both *R*^*2*^*y* and *Q*^*2*^ exceed 0.5, demonstrating the model's reliable explanatory and predictive capabilities. The OPLS-DA score plot shows distinct separation among sample points from the four process stages, with tight intra-batch clustering and no significant inter-stage overlap (Fig. 7B). Based on this model, six indicators (SYG, LQZGA, LQZGE, SYNZG, DDG, and VIS) with VIP > 1.0 were selected as quality differentiation markers for 15 batches of water extracts, water extract concentrates, ethanol precipitation supernatants, and ethanol precipitation concentrates (Fig. 7C). Of these, SYG and LQZGA were identified as key biomarkers (VIP > 2). To validate model reliability, 200 permutation tests were conducted, yielding a Q^2^ regression line Y‑intercept < 0, indicating no overfitting (Fig. 7D).

### Optimization of XECQ alcohol precipitation process and set up of design space

#### Screening of key process parameters

The results for the retention rates of terpenoid components (Y_1_), flavonoid components (Y_2_), phenethyl glycoside components (Y_3_), and solid removal rate (Y_4_) in each batch of intermediates under the Plackett–Burman experimental design. The interaction binomial models established through stepwise regression analysis demonstrated excellent predictive capability for all four response variables, with adjusted determination coefficients (Adj *R*^*2*^) of 0.9971 (Y_1_), 0.9542 (Y_2_), 0.9699 (Y_3_), and 0.9326 (Y_4_) (Table 6 and Table S9). Detailed regression equations for the models are provided in Supplementary Material S3.

**Table 6:** Plackett–Burman design linear regression results

Return type	Terpenoid retention rate (Y_1_)		Flavonoid retention rate (Y_2_)		Retention rate of phenethyl alcohol glycosides (Y_3_)		Solid removal rate (Y_4_)	
	Adj *R*^2^	*F*	Adj *R*^2^	*F*	Adj *R*^2^	*F*	Adj *R*^2^	*F*
Linear	0.7102	4.85	0.5784	3.16	0.7231	5.1	0.8046	7.47
Reduced linear	0.8234	13.82	0.5418	5.34	0.6958	9.39	0.773	10.37
Reduced 2FI	0.9971	415.81	0.9542	33.72	0.9699	45.32	0.9326	26.37

The results of the analysis of variance (ANOVA) (Table 7) indicate that there are significant differences in the effects of various process parameters on the response values. The solid content of the concentrate (A) exhibits a highly significant positive effect on Y_1_, Y_2_, and Y_3_ (*P* < 0.01). The ethanol volume fraction (B) was a highly significant factor for Y_1_, Y3 and Y_4_ (*P* < 0.01), while also exhibiting a highly significant effect on Y3 (*P* < 0.01). The ethanol content at the endpoint of alcohol precipitation (C) showed a highly significant positive effect on Y_1_, Y_2_ and Y_4_ (*P* < 0.01). In addition, multiple interactions (such as AC, AD, etc.) were identified as significant or extremely significant.

**Table 7:** Regression coefficients and analysis of variance for stepwise regression models

Project	Terpenoid retention rate (Y_1_)		Flavonoid retention rate (Y_2_)		Retention rate of phenethyl alcohol glycosides (Y_3_)		Solid removal rate (Y_4_)		Sum of absolute values of partial regression coefficients
	Coefficient	*P*-value	Coefficient	*P*-value	Coefficient	*P*-value	Coefficient	*P*-value	
A	2.568	0.001^**^	3.444	0.001^**^	3.361	0.002^**^	0.371	0.000^**^	9.743
B	0.471	0.008^**^	−0.145	0.054	0.079	0.006^**^	−0.402	0.008^**^	1.097
C	−0.019	0.001^**^	1.296	0.003^**^	0.945	0.079	0.249	0.002^**^	2.509
D	−0.243	0.455	−0.181	0.020^*^	0.031	0.043^*^	–	–	0.455
E	–	–	–	–	–	–	−0.107	0.217	0.107
F	0.127	0.005^**^	−0.171	0.003^**^	−0.338	0.004^**^	–	–	0.636
G	–	–	–	–	–	–	0.119	0.017^*^	0.119
AB	−0.025	0.011^*^	–	–	−0.012	0.190	–	–	0.037
AC	−0.014	0.032^*^	−0.065	0.005^**^	−0.041	0.011^*^	–	–	0.120
AD	0.010	0.004^**^	0.009	0.033^*^	–	–	–	–	0.019
AF	−0.008	0.020^*^	–	–	0.008	0.089	–	–	0.016
BE	–	–	–	–	–	–	0.001	0.010^*^	0.001
Adj *R*^*2*^	0.9971		0.9542		0.9699		0.9326		–

^*^Significant effect items, ^*^P < 0.05 indicates significant, ^**^P < 0.01 indicates highly significant

Comparison of the absolute partial regression coefficients of significant factors showed that factor A (concentrate solid content) exerted the strongest effect, followed by factor C (endpoint alcohol content) and factor B (ethanol volume fraction). Based on significance (*P*-values) and effect magnitude, factors A, B, and C were identified as key process parameters for subsequent Box-Behnken design optimization.

#### Establishment of mathematical models for key process units

Box-Behnken experimental design and results are conducted. Through stepwise regression analysis, simplified cubic models for the retention rates of terpenoids (Y_1_), flavonoids (Y_2_), and phenethyl glycosides (Y_3_), as well as a simplified quadratic model for solid removal rate (Y_4_), were established. Analysis of variance indicated that all models were highly significant (*P* < 0.05) with non-significant misfit terms (*P* > 0.05) (Table S10). The adjusted coefficient of determination (Adj *R*^*2*^) for each model exceeded 0.85 (Table 8), demonstrating good model fit to experimental results and strong predictive capability.

**Table 8:** Regression analysis results for active ingredient retention rate and solid removal rate models

Project	Terpenoid retention rate (Y_1_)		Flavonoid retention rate (Y_2_)		Retention rate of phenethyl alcohol glycosides (Y_3_)		Solid removal rate (Y_4_)	
	Coefficient	*P*-value	Coefficient	*P*-value	Coefficient	*P*-value	Coefficient	*P*-value
Constant	8076.985	–	5853.224	–	3600.731	–	−137.112	–
A	62.954	0.9469	−222.721	0.0021^**^	7.726	0.5112	−0.783	< 0.0001^**^
B	−259.720	0.0010^**^	−146.667	0.9195	−46.684	0.0148^*^	3.614	< 0.0001^**^
C	−35.991	0.3222	20.133	0.0015^**^	−107.944	0.0031^**^	−0.196	< 0.0001^**^
AB	–	–	6.075	0.3474	–	–	–	–
AC	−1.788	0.0008^**^	−1.174	0.0098^**^	−0.104	< 0.0001^**^	0.018	0.0878
BC	3.221	0.0068^**^	−0.057	0.0314^*^	1.331	0.0029^**^	–	–
A^2^	−0.041	0.0163^*^	−0.289	0.0352^*^	−0.019	0.1108	–	–
B^2^	1.690	0.0097^**^	0.792	0.1003	0.027	0.0392^*^	−0.019	0.0740
C^2^	−1.245	0.0330^*^	–	–	0.882	0.0002^**^	–	–
A^2^B	–	–	−0.013	0.0579	–	–	–	–
AB^2^	–	–	−0.030	0.0025^**^	–	–	–	–
AC^2^	0.013	0.0113^*^	–	–	–	–	–	–
A^2^C	–	–	0.022	0.0097^**^	–	–	–	–
B^2^C	−0.025	0.0007^**^	–	–	–	–	–	–
BC^2^	0.010	0.0328^*^	–	–	−0.011	0.0105^*^	–	–
Overall model	0.0011^**^		0.0072^**^		< 0.0001^**^		< 0.0001^**^	
Lack of fit	0.6115		0.6321		0.6474		5.8100	
Adj *R*^2^	0.9446		0.8786		0.9481		0.9411	

^*^Significant effect items, ^*^P < 0.05 indicates significant, ^**^P < 0.01 indicates highly significant

**Fig. 8:**
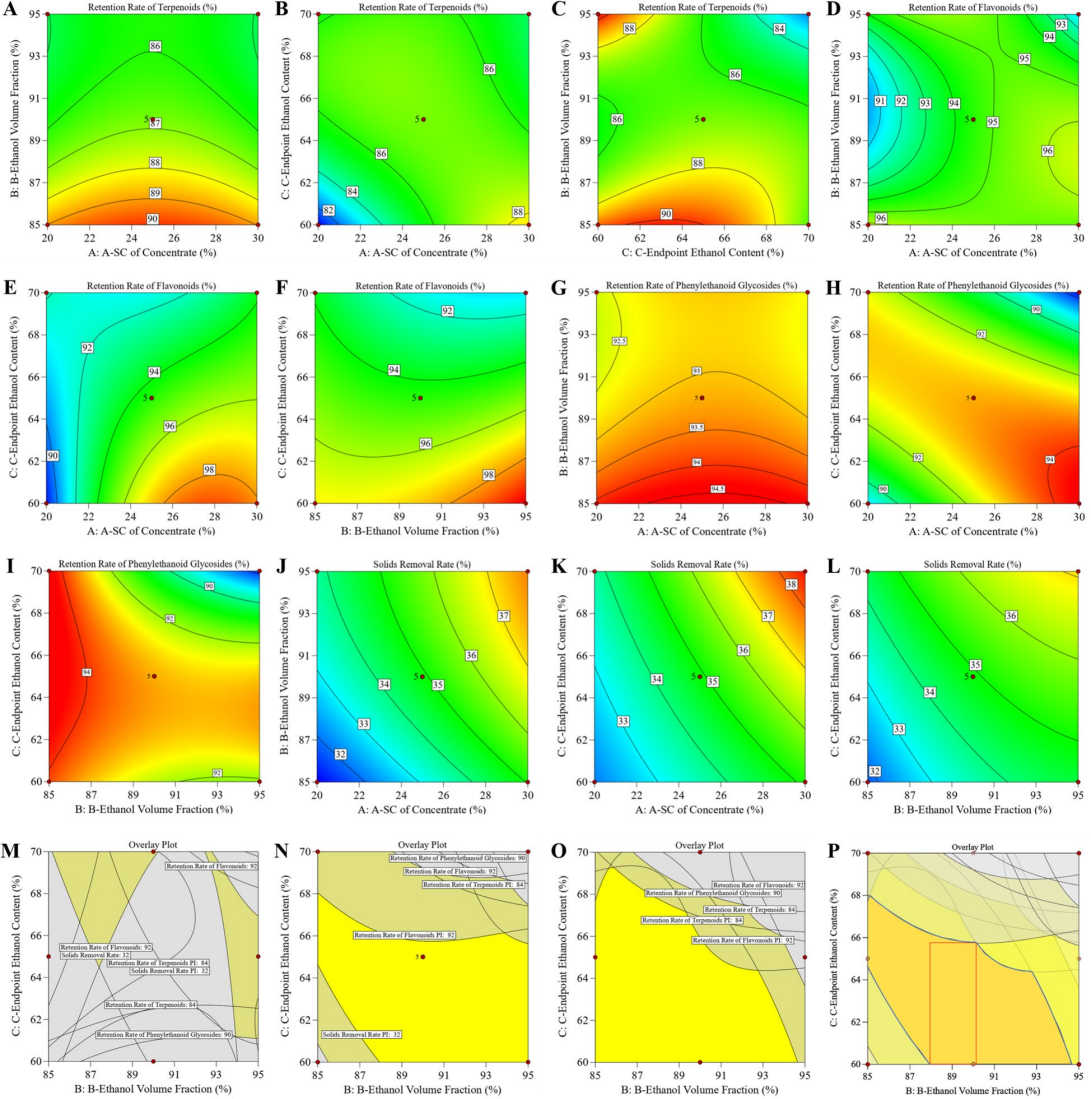
Response surface contour plots and design space for ethanol precipitation process parameters. **A**–**L** Response surface contour plots for retention rate and removal rate of various indicators. **A**–**C** Retention rate of terpenoid components (Y_1_). **D**–**F** Retention rate of flavonoid components (Y_2_). **G**–**I** Retention rate of phenethyl alcohol glycoside components (Y_3_). **J**–**L** Solid removal rate Y_4_. Among these, **A**, **D**, **G**, **J** fixed the final ethanol concentration at 65%. **B**, **E**, **H**, **K** fixed the ethanol volume fraction at 90%. **C**, **F**, **I**, **L** fixed the solid content of the concentrate at 25%. **M** Design space at 20% solid content in the concentrate. **N** Design space at 25% solid content in the concentrate. **O** Design space at 30% solid content in the concentrate. **P** Design space within the 25–30% solid content range in the concentrate. Blue irregular region represents the original feasible space; red boxed region indicates the experimental points selected for design space verification

Response surface analysis revealed complex interactions among key process parameters (Fig. 8A–L). The retention rate of terpenoid components (Y_1_) increased at lower ethanol volume fractions when the final ethanol concentration was ≥ 65%. In contrast, at lower final ethanol concentrations, the retention rate initially increased and then decreased with increasing ethanol volume fraction. Increasing solids content in the concentrate enhanced retention. However, beyond a certain final ethanol concentration, further increases in solids content produced negligible gains and could even reduce retention. At lower final ethanol concentrations combined with higher solids content, the retention rate of flavonoid components (Y_2_) increased. In contrast, under high solids content, elevated ethanol concentrations were associated with reduced retention.

A lower ethanol volume fraction enhanced the retention of phenethyl glycosides (Y_3_). Accordingly, when solids content was high, a reduced final ethanol concentration favored component retention, whereas at lower solids content, a moderate increase in final ethanol concentration improved retention. The solid removal rate (Y_4_) showed a positive correlation with all three process parameters.

#### Establishing the design space for alcohol precipitation process

Based on the Box–Behnken design, minimum acceptable criteria for key quality attributes were established to ensure intermediate quality (Table 9). Robust operating ranges were subsequently defined using a multi-indicator superposition approach combined with 95% prediction intervals.

**Table 9:** Control thresholds for active ingredient retention rate and solid removal rate

Indicator	Target control lower limit (%)
Terpenoid retention rate	84.0
Flavonoid retention rate	92.0
Retention rate of phenethyl alcohol glycosides	90.0
Solid removal rate	32.0

As shown in Fig. 8M–O, the design space was evaluated for concentrate solids contents of 20.0%, 25.0%, and 30.0%, respectively. It was observed that a suitable parameter space could not be formed at a solids content of 20.0%. Therefore, the control range for concentrate solids content was set to 25.0–30.0%. Since the resulting design space was irregular, the spaces corresponding to solids contents of 25.0–30.0% were overlapped to define a rectangular area for practical process operation, as shown in Fig. 8P. This region was designated as the design space for the XECQ ethanol precipitation process, with the following parameter ranges: concentrate solids content of 25.0–30.0%, ethanol volume fraction of 88.0–90.0%, and final ethanol concentration of 60.0–65.7%.

#### Establishing control spaces

Validation points were selected both inside and outside the control space (Table 10). Points T_1_–T_3_ represented experimental points within the design space, with control parameters set at 30.0% concentrate solids content, 90.0% ethanol volume fraction, and 60.0% final ethanol concentration. The results indicated that the RSD values for the measured responses after ethanol precipitation were 1.622%, 1.028%, 1.332%, and 3.086%. Test points T_4_–T_6_, located outside the control space, failed to meet the control targets, confirming that the predictive capability of the defined control space was satisfactory.

**Table 10:** Control thresholds for active ingredient retention rate and solid removal rate

Test	A (%)	B (%)	C (%)	Y_1_ (%)	Y_2_ (%)	Y_3_ (%)	Y_4_ (%)
Inside T_1_ space	30	90	60	90.322	95.768	93.313	38.358
Inside T_2_ space	30	90	60	90.150	96.442	93.446	36.107
Inside T_3_ space	30	90	60	87.729	94.506	91.244	36.869
Outside T_4_ space	25	85	60	88.991	96.075	93.988	31.842
Outside T_5_ space	30	95	70	82.719	88.892	88.631	40.294
Outside T_6_ space	20	90	60	86.282	92.049	89.124	32.241

### Machine vision-based evaluation method for XECQ quality consistency

#### Color feature extraction and visualization of XECQ digital images

**Fig. 9:**
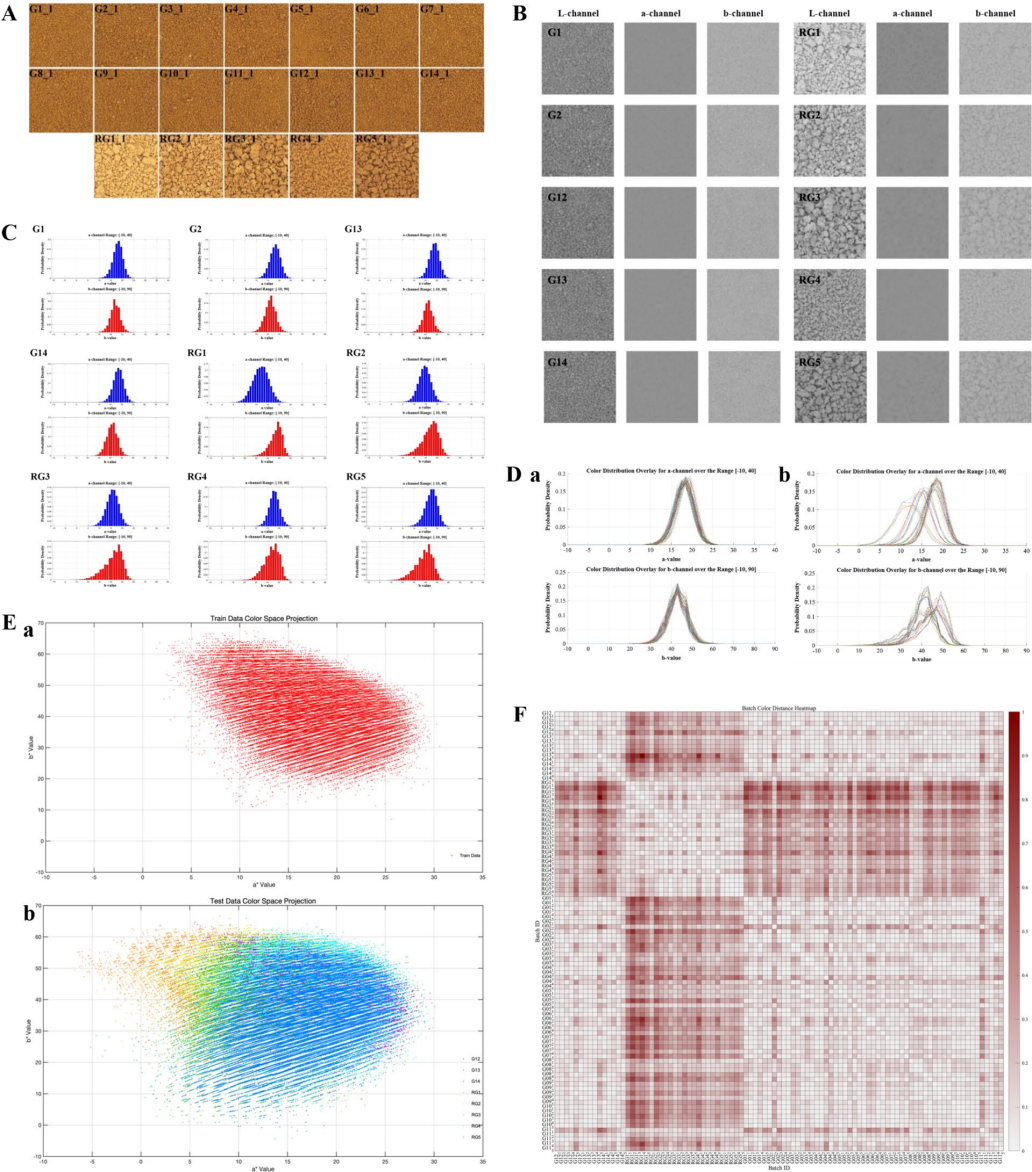
Analysis of visual attributes and color characteristics of XECQ particles. **A** Image representation of XECQ particles. G1_1-G14_1: XECQ standard particles. RG1_1-RG5_1: Custom-made XECQ reference particles (400 × 400 pixels). **B** Visualized L, a, b channels of XECQ particle images. **C** Histograms of a and b chromaticity distributions for XECQ particle images. **D** Overlaid histograms of a and b chromaticity distributions for XECQ particle training set (a) and test set (b) images. **E** Color space projections of XECQ particle training set (a) and test set (b) images in the a/b channel. **F** Heatmap of color distance between XECQ particle batches

To quantitatively evaluate the color consistency of XECQ particles, sub-images of the central regions (Fig. 9A) were analyzed for standard particles (G1–G14) and reference particles (RG1–RG5). The images were converted from RGB to Lab color space to separate luminance (L) and chromaticity (a, b) information. Chromaticity distributions in the a/b channels differed markedly between standard and reference particles. Normalized probability density histograms of the central region sub-images across batches showed that standard particles exhibited more concentrated a/b distributions with consistent patterns across batches, whereas reference particles exhibited greater variability (Fig. 9B, C). Overlaying histograms of all training and testing samples revealed that the color distributions of standard particles were highly concentrated, while those of reference particles were more dispersed, indicating lower color consistency (Fig. 9D).

To avoid potential information loss from histogram binning, color projection was employed for dimensionality reduction in visualization. This method deconstructed image color information into discrete point clouds, allowing intuitive visualization of chromaticity distribution characteristics across particle images from different batches (Fig. 9E). The results indicated that the chromaticity point cloud clusters of standard particles and training set particles exhibited similar aggregation patterns, whereas those of reference particles were more dispersed, with numerous outliers along the edges.

Additionally, global differences across all batches were quantified using color distance heatmaps (Fig. 9F). Heatmaps among standard particle batches appeared predominantly white, indicating minimal color variation, whereas heatmaps between reference and standard particles appeared predominantly red, reflecting substantial color differences.

#### XECQ digital image texture and density feature extraction and visualization

**Fig. 10:**
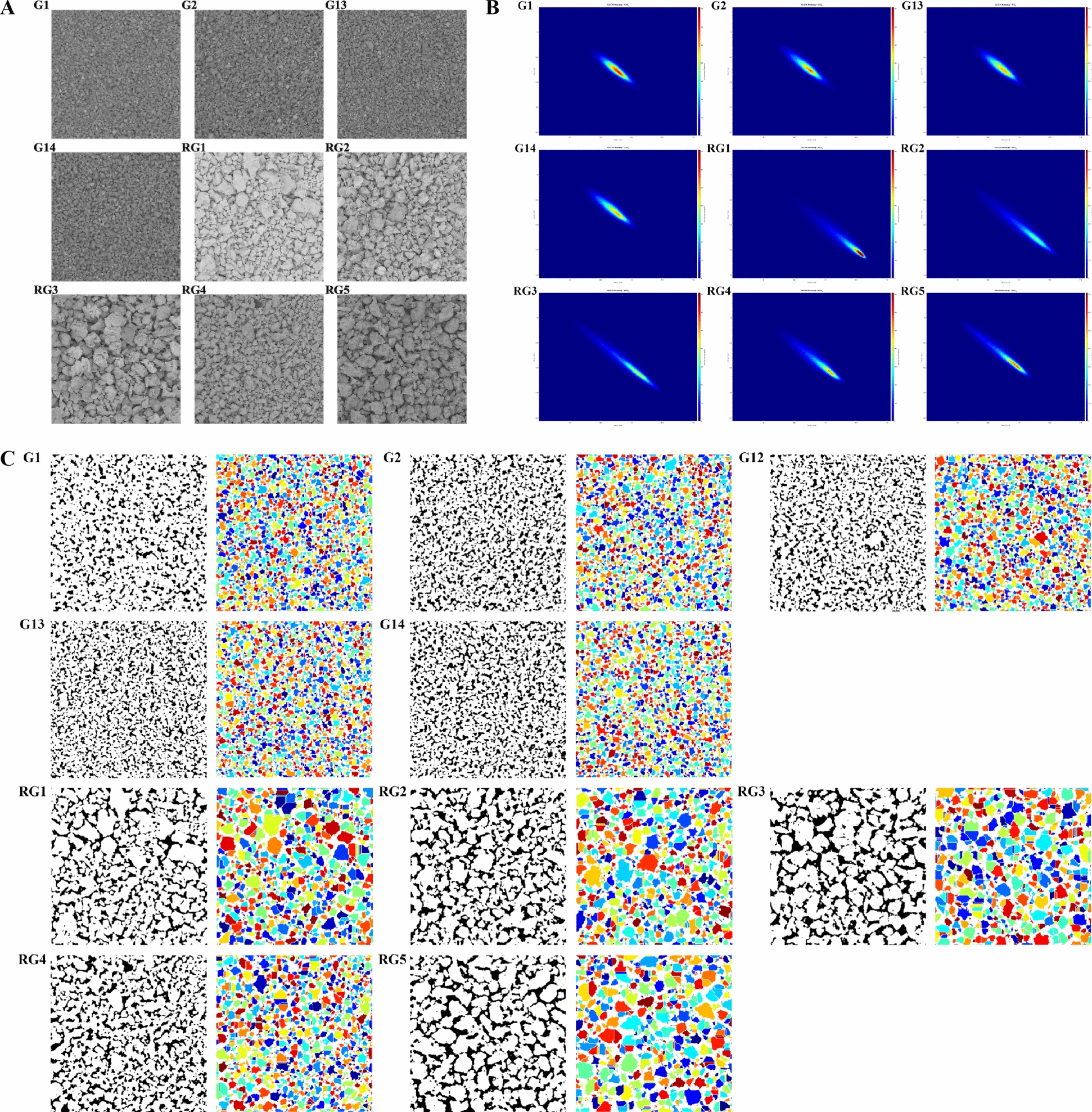
Analysis of texture and density characteristics of XECQ granules. **A** Grayscale image from the central region of the XECQ granule image. **B** Grayscale heatmap from the central region of the XECQ granule image. **C** Adaptive binarization and watershed segmentation results from the central region of the XECQ granule image

To systematically evaluate the microstructure of XECQ, images of the central regions of standard and reference particles were analyzed from both texture and spatial distribution perspectives. First, particle surface texture was quantified using the gray-level co-occurrence matrix (GLCM), with statistical analysis performed on four parameters: contrast, correlation, energy, and homogeneity. To visually illustrate differences in texture properties, normalized GLCM matrices were presented as heatmaps. Color intensity (blue → red gradient) intuitively represents the co-occurrence probability of different gray-level pairs, with redder colors indicating more frequent appearance of the corresponding gray-level combinations. Representative sub-images from the central regions of G1–G2, G12–G14, and RG1–RG5 batches were analyzed. Analysis of their grayscale images and corresponding GLCM heatmaps at 256 grayscale levels revealed that the highlighted regions (high-probability areas) in the standard particle heatmaps exhibited a spindle-shaped distribution concentrated along the diagonal (Table S11). This indicated highly similar grayscale values in adjacent pixels, reflecting consistent surface reflectance and a concentrated particle size distribution. In contrast, the bright regions in the GLCM heatmaps of reference particles exhibited clear shifts and dispersion (Fig. 10A, B).

Secondly, we segmented the central region sub-images of particles from batches G1-G2, G12-G14, and RG1-RG5 were segmented using adaptive binarization and watershed algorithms to quantify spatial distribution characteristics. As shown in Fig. 10C, quantitative analysis of particle spatial distribution, including particle density, number, mean area, and standard deviation of area, revealed significant differences between standard and reference particles (Table S12).

#### Particle quality assessment system with multi-dimensional feature fusion

The particle quality assessment system was run to establish a quality benchmark for XECQ granules based on training images (trained_params.mat). The test set was then evaluated, producing a feature matrix file (feature_matrix.xlsx) and a quality inspection report (quality_report.xlsx). Among the standard granule test samples, G12_3.jpg and G13_5.jpg triggered a review report due to their total scores falling within the range 85 ≤ *S*_*total*_ < 95, prompting quality control personnel to re-examine these results (Table S13). Analysis of the feature score modules indicates that G12_3.jpg scored lower than other samples in the same batch for color histogram and color feature statistics. This suggests that the mixture of granule extract and excipients in this area may be less uniform than in other regions. Meanwhile, G13_5.jpg exhibited abnormal texture feature integration scores, likely due to uneven particle size distribution in this area. For the reference particles, the report indicates all are “Reject”. Among them, RG4_5.jpg has a low total score but a Mahalanobis distance score of 0.968. Since Mahalanobis distance similarity depends on feature deviation from the mean, this indicates the distribution of this sample’s feature vector aligns with that of the training set feature vectors. Consequently, their covariance structures remain similar, resulting in a close Mahalanobis distance despite significant differences in raw feature values.

The system employed a weighted scoring and Mahalanobis distance-based anomaly detection strategy, providing accurate quality assessments. Compared with traditional powder testing methods, this machine vision system substantially improves the efficiency of particle appearance evaluation.

## Discussion

Quality consistency control of TCM compound preparations is a key bottleneck constraining their modernization and internationalization.

This study establishes a quality control system for XECQ that integrates deep machine learning, QbD-driven process optimization, and machine vision-based online monitoring. The system addresses quality control challenges while providing a scalable, data-driven framework and a research paradigm from theory to practice for navigating the traditional “black box” of complex TCM systems.

Through multidimensional analysis, this study first reveals the dynamic mechanism of synergistic co-evolution between “constituents and physical properties” during pretreatment. During the water extraction and concentration stage, the proportion of terpenoid constituents decreased significantly due to their heat sensitivity, whereas flavonoids and phenethyl glycosides were relatively enriched owing to their higher thermal stability and solubility. This finding extends traditional interpretations based primarily on polarity differences, highlighting the critical role of properties such as heat sensitivity and solubility in component evolution. More importantly, the study discovered a reversal in the correlation between “components-physical properties” before and after alcohol precipitation. Mechanistically, alcohol precipitation fundamentally alters the solvent polarity and intermolecular interaction network within the system. The introduction of high concentrations of ethanol decreases the dielectric constant of the medium, weakens hydration layers surrounding hydrophilic macromolecules (e.g., polysaccharides and proteins), and promotes their aggregation and precipitation. As these macromolecular colloidal components are major contributors to viscosity through hydrogen bonding, chain entanglement, and water retention, their removal substantially reduces the structural viscosity of the system. Simultaneously, the redistribution of small-molecule constituents (such as flavonoids, phenylethanoid glycosides, and terpenoids) occurs due to differential solubility under reduced polarity conditions. More polar components preferentially partition into the supernatant, whereas less soluble or weakly polar constituents may co-precipitate with macromolecular matrices. This selective partitioning reshapes the compositional balance between macromolecules and small molecules, thereby modifying intermolecular interaction patterns (e.g., hydrogen bonding, π–π stacking, and hydrophobic interactions). As a result, the dominant determinants of physical properties shift from macromolecular network structures to the relative proportions and interaction states of retained small molecules.

Therefore, the observed “reversal” in component–property correlations reflects a transition in the system’s structural hierarchy: before alcohol precipitation, viscosity and other physical parameters are primarily governed by colloidal macromolecular networks; after precipitation, these properties are influenced more by the composition and interaction state of the remaining soluble fractions. In essence, alcohol precipitation acts as a physicochemical reorganization process rather than a simple impurity removal step, reconstructing the internal interaction topology of the formulation. This explains why component redistribution not only reduced viscosity RSD (improving homogeneity) but also altered the direction and strength of component–property correlations. This indicates that the macroscopic physical parameters of TCM formulations are not a simple sum of individual component properties but rather complex interactions among all constituents. Consequently, adjustments to process parameters can affect physical properties in a nonlinear manner. This mechanism provides scientific rationale for indirectly predicting difficult-to-measure chemical quality attributes through easily measurable physical indicators, thereby establishing a theoretical foundation for the application of process analytical techniques in TCM production.

Based on the above, this study applied QbD to optimize the critical ethanol precipitation process [27]. Given the inherent variability of raw herbal materials, traditional fixed-parameter processes often fail to ensure stable production and consistent product quality. Through multi-objective response surface modeling, this study elucidated the complex interactions among the solid content of the concentrate, ethanol volume fraction, and final ethanol concentration, thereby establishing a design space that replaces conventional single-parameter settings.

This approach significantly enhances the process’s adaptability to raw material variations, propelling TCM production from an “experience-driven” to a “data-driven” paradigm. It paves the way for real-time release, achieving both efficient impurity removal and effective preservation—two critical objectives—in a balanced manner.

To address the delays inherent in chemical quality inspection, this study developed a machine vision–based intelligent detection system, marking an innovative shift in quality evaluation. By extracting 112 morphological and textural features and applying Mahalanobis distance for anomaly detection, the system enables quantitative, multi-scale analysis of appearance quality, with sensitivity sufficient to identify minute defects imperceptible to the human eye. This system forms a closed-loop quality control framework in conjunction with the QbD approach: QbD proactively ensures quality during process design, while machine vision provides rapid, non-destructive final inspection and grading at the product stage. It reduces inspection time from hours to real time, allowing online, unit-by-unit non-destructive testing, thereby overcoming the statistical limitations of traditional destructive sampling.

Of course, this study has certain limitations, which also point to clear directions for further research. Although the transfer patterns of components and physical properties were analyzed throughout the entire process from extraction to paste collection, the current quality control strategy focuses primarily on the precise optimization of the alcohol precipitation and subsequent steps. For the front-end extraction process, online real-time monitoring methods and closed-loop control strategies for key quality attributes have yet to be established. Future research could incorporate process analytical technologies (such as near-infrared spectroscopy, NIRS) to enable real-time monitoring of key quality attributes of extraction intermediates. Combined with model predictive control (MPC) algorithms, this could establish closed-loop strategies that transition from “static design space” to “dynamic real-time regulation”, ultimately achieving intelligent quality control throughout the entire process from raw materials to finished products. In machine vision, integrating convolutional neural networks (CNNs) for autonomous extraction of high-dimensional features and combining them with multispectral imaging technology to establish quantitative structure–activity relationships between external morphology and internal chemical composition will be a key direction for further enhancing the system’s discrimination capability and interpretability.

## Conclusion

This study integrates multidimensional quality transfer analysis, QbD-based process optimization, and intelligent machine vision monitoring to establish a comprehensive, data-driven quality control framework. This framework not only elucidates the dynamic coupling between composition and physical properties in XECQ and establishes a robust design space for the critical alcohol precipitation process, but also develops a non-destructive machine vision system for real-time quality monitoring. Its core innovation lies in achieving a synergistic closed loop between pre-production parameter optimization and post-production intelligent quality inspection, offering a new paradigm to address quality consistency challenges in complex natural product formulations and driving the intelligent transformation of TCM manufacturing.

## Supplementary Information

Supplementary material 1.

## Data Availability

Data will be made available on request.

## Abbreviations

XECQ: Xiaoer Chiqiao Qingre granules
TCM: Traditional Chinese medicine
QbD: Quality by design
HPLC: High performance liquid chromatography
CQAs: Critical quality attributes
ADME: Absorption, distribution, metabolism, and excretion
QbT: Quality by testing
QTPP: Quality target product profile
CMAs: Critical material attributes
CQAs: Critical quality attributes
CPPs: Critical process parameters
DoE: Design of experiments
YHSYG: Oxypaeoniflorin
SYG: Paeoniflorin
MSZXSYG: Galloylpaeoniflorin
SYNZG: Paeonolide
MBHG: Ononin
DDG: Daidzin
RLMS: Genistein
HHQG: Wogonoside
LQZGA: Forsythoside A
LQZGE: Forsythoside E
HJTG: Salidroside
SG: Specific gravity
SC: Solid content
VIS: Viscosity
*σ*: Conductivity
RI: Refractive index
PCA: Principal component analysis
OPLS-DA: Orthogonal partial least squares discriminant analysis
HB_0_: Mixed standard solutions
RSD: Relative standard deviation
LOD: Limit of detection
S/N: Signal-to-noise ratio
LOQ: Limit of quantification
RRT: Relative retention time
RPA: Relative peak areas
ANOVA: Analysis of variance
GLCM: Gray-level co-occurrence matrix
NIRS: Near-infrared spectroscopy
MPC: Model predictive control
CNNs: Convolutional neural networks
