# Supplementary material for: Data-driven full-chain quality control for Xiaoer Chiqiao Qingre granules: from process risk management to intelligent product testing
Source: Chin Med. 2026 May 12;21:130. doi: 10.1186/s13020-026-01414-z (PMC13162461; doi:10.1186/s13020-026-01414-z)
Supplement: Supplementary file 1 — Supplementary material 1. [file 13020_2026_1414_MOESM1_ESM.docx]

**Data-driven full-chain quality control for Xiaoer Chiqiao Qingre granules: From process risk management to intelligent product testing**

Nan Wang^1#^, Yukun Chang^1#^, Yue Cheng^1^, Jing Zhao^2^, Chao Li^2^, Lu Sun^1,3^, Liang Feng^1, 2, 3*^, Yanjun Yang^1, 2*^, Xiaobin Jia^1, 2*^

^1^ Jiangning Hospital of Chinese Medicine, School of Traditional Chinese Pharmacy, China Pharmaceutical University, Nanjing 211198, P.R. China

^2^ Jiangsu Key Laboratory of Chinese Medicine and Characteristic Preparations for Paediatrics, Jumpcan Pharmaceutical Co., Ltd., Taixing 225400, P.R. China

^3^ Nanjing Jiangning District Hospital of Chinese Medicine, Nanjing 211100, P.R. China

^#^**First authors:** Contributed equally to this work

^*^**Corresponding authors**

Xiaobin Jia, Nanjing 211198, P.R. China. E-mail address(es): jiaxiaobin2015@163.com, Tel./fax: +86-13605157558.

Yanjun Yang, Nanjing 211198, P.R. China. E-mail address(es): 15706037600@163.com, Tel./fax: +86-15706037600.

Liang Feng, Nanjing 211100, P.R. China. E-mail address(es): wenmoxiushi@163.com,

Tel./fax: +86-13951898629.

**Table of Contents**

1. **Table S1** Calibration curves, regression equations, linear ranges, and correlation coefficients of 11 indicator components
2. **Table S2** Stability test results
3. **Table S3** Limit of detection (LOD) and quantitation (LOQ) for 11 types of components in XECQ
4. **Table S4** Accuracy assessment of selected components based on sample recovery experiments conducted at three spiking levels
5. **Table S5** Quantitative determination of 11 indicator components in water extracts from different batches
6. **Table S6** Quantitative determination of 11 indicator components in water extract concentrates from different batches
7. **Table S7** Quantitative determination of 11 indicator components in the supernatant of alcohol precipitation from different batches
8. **Table S8** Quantitative determination of 11 indicator components in alcohol precipitation concentrates from different batches
9. **Table S9** Plackett-Burman design and responses for terpenoid, flavonoid, phenethyl glycoside retention rates, and solid removal rate
10. **Table S10** Box-Behnken experimental design and results for key process units
11. **Table S11** Digital image texture features of XECQ particles extracted by GLCM analysis
12. **Table S12** Spatial distribution characteristics of standard and reference XECQ granules
13. **Table S13** Particle quality assessment results of XECQ granules based on multi-dimensional feature fusion
14. **Figure S1** Fishbone diagram for risk analysis of XECQ preparation process
15. **Figure S2** HPLC chromatograms of mixed standard solution
16. **Figure S3** HPLC chromatograms of test samples
17. **Figure S4** Structural framework of the particle quality assessment system

**Supplementary Material S1**

**1. Evaluation criteria and risk analysis for alcohol precipitation process**

In the production of TCM formulations, alcohol precipitation serves as a core unit operation with dual effects: while effectively reducing impurity content, it may also lead to loss of active ingredients. Consequently, its process performance directly impacts the safety and efficacy of the final product. Therefore, process research must simultaneously evaluate the dynamic equilibrium between key parameters and the retention rate of target active substances alongside impurity removal efficiency. The retention efficiency of active ingredients can be characterized by the effective component retention rate:

$$R_{a}=\frac{m_{as}}{m_{ac}}\times100\% (Formula S1)$$

Here, *R_a_* represents the retention rate of active ingredients, *m_as_* denotes the mass of active ingredients in the alcoholic precipitation supernatant, and *m_ac_* indicates the mass of active ingredients in the water extract concentrate. A higher retention rate indicates less loss of active ingredients during the alcoholic precipitation process.

The removal efficiency of impurities by the alcohol precipitation process is expressed as the solid removal rate, calculated using the following formula:

$$R_{s}=\frac{m_{ss}}{m_{sc}}\times100\% (Formula S2)$$

Where *R_s_* denote the solid removal rate, *m_ss_* represent the mass of solids precipitated during alcohol precipitation, and *m_sc_* denote the mass of solids in the water-extracted concentrate.

Using terpenoid retention rate, flavonoid retention rate, phenethyl glycoside retention rate, and solid removal rate as process performance indicators, calculations are performed according to **Formula S1** and **Formula S2**. A fishbone diagram for risk analysis of the XECQ preparation process (**Figure S1**) was constructed based on a literature review spanning 2014–2024, identifying process parameters with potential risks during the alcohol precipitation process.


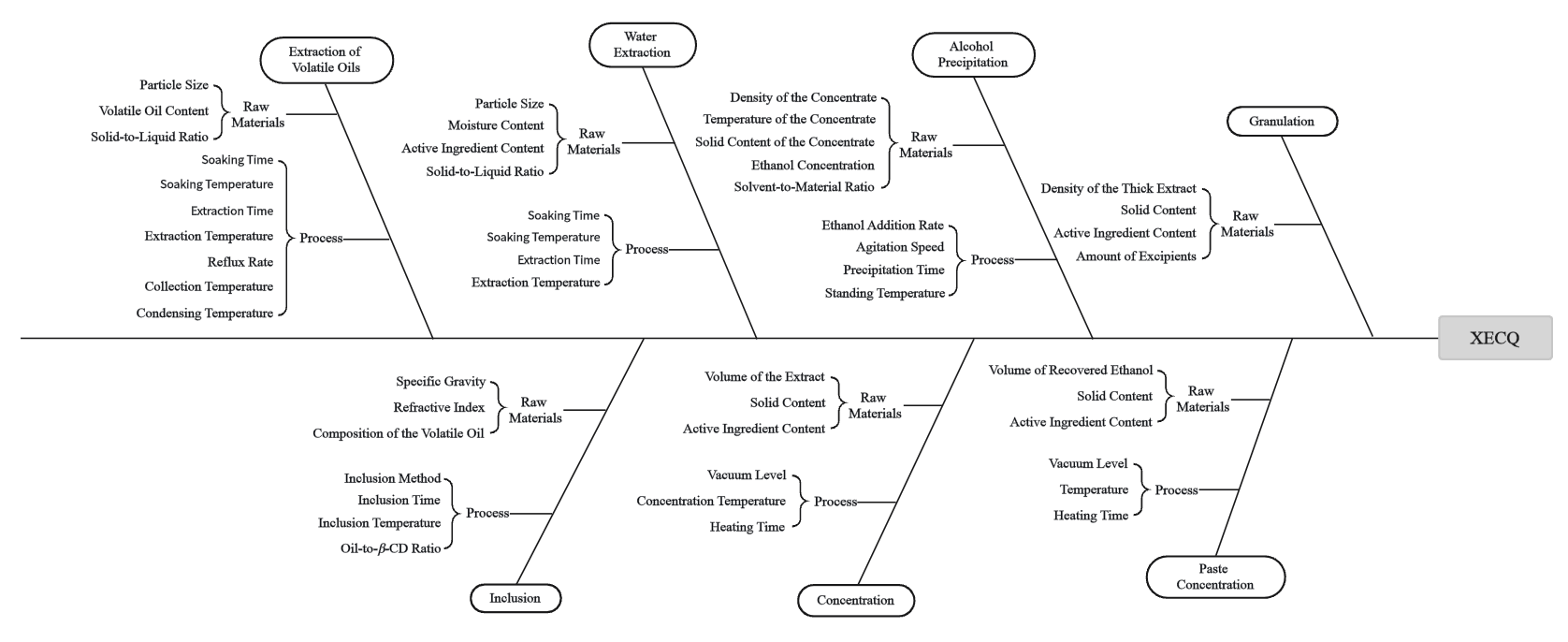


**Figure S1.** Fishbone diagram for risk analysis of XECQ preparation process.

**Supplementary Material S2**

**2.1 Method for constructing a digital image feature extraction and quality assessment model**

*2.1.1 Extraction of color features from digital images*

In particle quality analysis, the human eye is sensitive to variations in color depth, uniformity, and void color. Compared to the light sensitivity of RGB space and the color gamut limitations of HSV space in industrial applications, the Lab color space offers significant advantages in perceiving color uniformity. Its decoupled luminance (L) and chroma (a, b) channels are better suited for quantifying subtle color differences. To address this, the system adopts the CIE-Lab color space, separating the luminance (L) channel from the chromaticity (a, b) channels. Here, a represents red-green axis color differences, while b represents yellow-blue axis color differences. This space aligns closely with human visual perception, significantly enhancing the physical interpretability of color features.

Input standard RGB images are converted to Lab color space via nonlinear mapping to extract chromaticity values from the a and b channels. In training mode, binning parameters are generated based on standard batch data, performing uniform binning statistics on pixel values in the a and b channels (50 intervals each). The dynamic range is set to a ∈ [-10, 40] and b ∈ [-10, 90], covering the chromaticity distribution of typical particle samples. The normalized probability density histogram is computed via frequency statistics and interval smoothing, using the formula:

$$P_{channel}\left( x \right)=\frac{\sum I{(x}_{i}\in{bin}_{k})}{N_{pixel}}, {bin}_{k}\in\left[ L_{min},L_{max} \right] (Formula S3)$$

Where *I* denote the indicator function, *N_pixel_* represents the total number of pixels in the channel, and [*L_min_*, *L_max_*] denote predefined dynamic boundaries. The same parameters are reused during testing to ensure cross-batch feature consistency. The normalized histogram probability distributions for channels a and b (50 dimensions each) are obtained. Combined with the global mean and standard deviation (4 dimensions), they form the color feature set (104 dimensions total).

*2.1.2 Digital image texture feature extraction*

The uniformity and regularity of particle surfaces can be quantitatively characterized through texture features. The GLCM effectively captures microstructural variations by statistically analyzing the co-occurrence probability of gray levels.

Convert RGB images to single-channel grayscale images. Calculate the joint probability distribution of grayscale value pairs in the horizontal direction (*θ*=0°) to construct the matrix *P*(i,j), where *i* and *j* represent the grayscale values of adjacent pixels, and the matrix element values denote the frequency of occurrence for that grayscale pair. Based on *P*(i,j), four statistical measures are calculated: Contrast, correlation, energy, and homogeneity. These respectively describe texture clarity, directional consistency, intensity distribution, and local similarity, forming a complementary feature set (4-dimensional total). The calculation formulas are as follows:

$$Contrast=\sum\left| i-j \right|^{2}P\left( i,j \right) \left( Formula S4 \right)$$

$$Correlation=\sum\frac{\left( i-\mu_{i} \right)\left( j-\mu_{j} \right)P(i,j)}{\sigma_{i}\sigma_{j}} (Formula S5)$$

$$Energy=\sum P{(i,j)}^{2} (Formula S6)$$

$$Homogeneity=\sum\frac{P(i,j)}{1+\left| i-j \right|} (Formula S7)$$

*2.1.3 Extraction of digital image density features*

Particle density and morphology directly influence quality assessment outcomes. Traditional binarization methods are susceptible to noise and agglomerated particles. Adaptive thresholding dynamically perceives local texture features, adjusting segmentation boundaries according to particle size and distribution density to significantly enhance contour integrity in heterogeneous particle clusters. This system employs adaptive threshold segmentation for image binarization, combined with morphological opening operations to eliminate noise interference. It utilizes the Watershed Algorithm for particle segmentation and calculates particle density, count, mean area, and standard deviation (4 dimensions) to characterize density features. The implementation workflow is as follows:

(1) Image grayscaling and binarization processing: After converting the input RGB image to grayscale (using the im2gray function), the imbinarize function is applied to segment the granular regions through adaptive thresholding. The threshold T(x,y) is calculated as follows:

$$T\left( x,y \right)=\mu\left( x,y \right)-\alpha\cdot\sigma\left( x,y \right) (Formula S8)$$

Here, μ(x,y) and σ(x,y) represent the mean and standard deviation of the local window centered at pixel (x,y) (default size 51×51), respectively, while *α* denotes the sensitivity parameter. Subsequently, a morphological opening operation (imopen function) is performed using a circular structural element with a fixed pixel radius to eliminate isolated noise points and smooth particle edges.

Sensitivity determines the strictness of threshold segmentation. Higher sensitivity corresponds to a lower threshold, resulting in more pixels being classified as foreground (grain regions). Radius represents the size of the circular structural element in morphological opening operations. A larger radius enhances noise suppression but may cause excessive erosion of fine grains. Based on the overall binarization results, the parameters were set to Sensitivity=0.7 and Radius=1.

(2) Watershed divide: Calculate the negative euclidean distance (NED) of background regions in a binary image, generate a distance field matrix D, and label non-particle regions (background) as -∞. This forces the watershed algorithm to process only particle regions. Apply watershed transformation to segment the image into independent connected components, outputting a label matrix L where background labels are 0 and particle labels start incrementing from 1.

(3) Regional attribute statistics: Based on the label matrix L, the regionprops function is used to extract the particle area attribute and compute the following features:

Particle density (Density): The ratio of the total effective particle area to the image resolution (Formula S9).

Particle count (N): Count the number of non-zero unique labels (Formula S10) to avoid background interference.

Area statistics: Calculate the mean area (Mean Area) (Formula S11) and standard deviation (Std Area) (Formula S12) of particle areas.

$$Density=\frac{\sum_{k=1}^{N} {Area}_{k}}{H\times W} (Formula S9)$$

$$N=numel (unique(L (L>0))) (Formula S10)$$

$$Mean Area=\frac{1}{N}\sum_{k=1}^{N} {Area}_{k} (Formula S11)$$

$$Std Area=\sqrt{\frac{1}{N-1}\sum_{k=1}^{N} {{(Area}_{k}-Mean Area)}^{2}} (Formula S12)$$

*2.1.4 Quality assessment model for multi-dimensional feature fusion*

This model achieves granular quality assessment through multidimensional feature fusion and a dynamic decision mechanism. Its core workflow comprises four key steps: feature fusion modeling, dynamic parameter learning, hierarchical scoring calculation, and adaptive decision-making. The specific methodology is as follows:

(1) Feature fusion and normalization: After extracting 112-dimensional feature vectors based on the Lab color space, a hybrid normalization strategy is employed to achieve dimensional consistency and comparability in multimodal feature construction. For color histogram features (50 dimensions each for the a/b channels), the Bhattacharyya coefficient quantifies the similarity between the distribution of test samples and the training set:

$${BC}_{i}=\sum_{k=1}^{50} \sqrt{P_{k}^{(i)}}\text{∙}q_{k}\times50 (Formula S13)$$

Here, $P_{k}^{(i)}$denotes the normalized pixel frequency of the *k*-th bin for the *i*-th sample, while *q_k_* represents the mean of the corresponding bin in the training set. Cross-sample histogram matching is achieved through bin parameter alignment.

Color statistical features (mean and standard deviation of a/b channels) along with texture and density features were normalized using a dynamic safe interval approach, with adaptive thresholds constructed based on the distribution characteristics of the training set: Features conforming to normal distribution (Anderson-Darling test *P* > 0.05) employed 3*σ* boundaries: [*μ*−3*σ*, *μ*+3*σ*], covering 99.73% of data as expected by statistical theory. Non-normally distributed features utilized quantile-truncated for extreme values: [*Q_0.01_*, *Q_0.0_*_9_], preventing interval distortion caused by incorrect distribution assumptions. This design balances the rigor of statistical assumptions with the complexity of real-world data, avoiding overfitting caused by a single threshold.

(2) Hierarchical scoring mapping mechanism: The mapping process converting raw feature values into quality scores comprises two stages: Histogram similarity score: Bhattacharyya coefficient linearly mapped to a 0–100 score (50 points each for channels a and b), reflecting the consistency of color distribution with the training set.

Feature compliance score: For statistical, textural, and density features, apply an exponentially decaying score based on boundary distance:

$$S_{j}(x)=25\times max(e^{-0.7\frac{\left| x-B \right|}{\sigma_{j}}},{10}^{-3}) (Formula S14)$$

In the formula, *B* represents the nearest boundary point, σ_j_ denotes the standard deviation of the training set, and the exponential decay coefficient 0.7 is determined through grid search optimization to achieve a balance between sensitivity and fault tolerance.

(3) Multi-dimensional score fusion: Module weight allocation is a weighted fusion of color histogram (20%), color statistical features (20%), texture (30%), and density (30%):

*S_total_* represents the total feature score after fusion; *S_hist_* denotes the color histogram distribution, reflecting the similarity of particle chromaticity distribution; *S_stats_* indicates the color mean and standard deviation statistics, reflecting the uniformity of particle chromaticity distribution; *S_texture_* is the texture feature score, characterizing the regularity of particle arrangement and sensitivity to minute defects; *S_density_* is the density feature score, characterizing the distribution of particles.

(4) Dynamic anomaly detection and decision-making: To overcome the linear weighting's disregard for feature correlations, the Mahalanobis distance metric is introduced to measure the overall deviation of the feature space:

$$D_{M}(X)=(X-\mu)\sum^{+}({X-\mu)}^{T} (Formula S15)$$

Σ^+^ denotes the Moore-Penrose inverse of the covariance matrix, addressing the issue of matrix singularity caused by small sample sizes. By calculating the 95th percentile *θ_D_* of the training set *D_M_*, a dual-threshold decision rule is established:

When S_total_ ≥ 95 and *D_M_* < *θ_D_*, the result is deemed acceptable (Accept);

When 85 ≤ S_total_ < 95 or *D_M_* ≥ *θ_D_*, manual review is triggered (Review);

When S_total_ < 85 or the reviewed sample's *D_M_* ≥ *θ_D_*, the result is deemed defective (Reject).

This model eliminates dimensional differences through feature layer normalization, balances contributions from various modalities using weighted fusion, and achieves multi-level fusion and dynamic threshold decision-making by integrating statistical distributions with spatial distances.

**Supplementary Material S3**

**3.1 Regression equation for the interactive binomial model**

$Y_{1}=\text{61.965+2.568A+0.471B-0.019C-0.243D+0.127F-0.025AB-0.014AC+0.010AD-0.008AF}$ ($Formula S16$)

$Y_{2}=\text{43.135+3.444A-0.145B+1.296C-0.181D-0.171F-0.065AC+0.009AD}$ $(Formula S17)$

$Y_{3}=\text{32.330+3.361A+0.079B+0.945C+0.031D-0.338F-0.012AB-0.041AC+0.008AF}$ $(Formula S18)$

$$Y_{4}=\text{41.323+0.371A-0.402B+0.249C-0.107E+0.119G+0.001BE} (Formula S19)$$

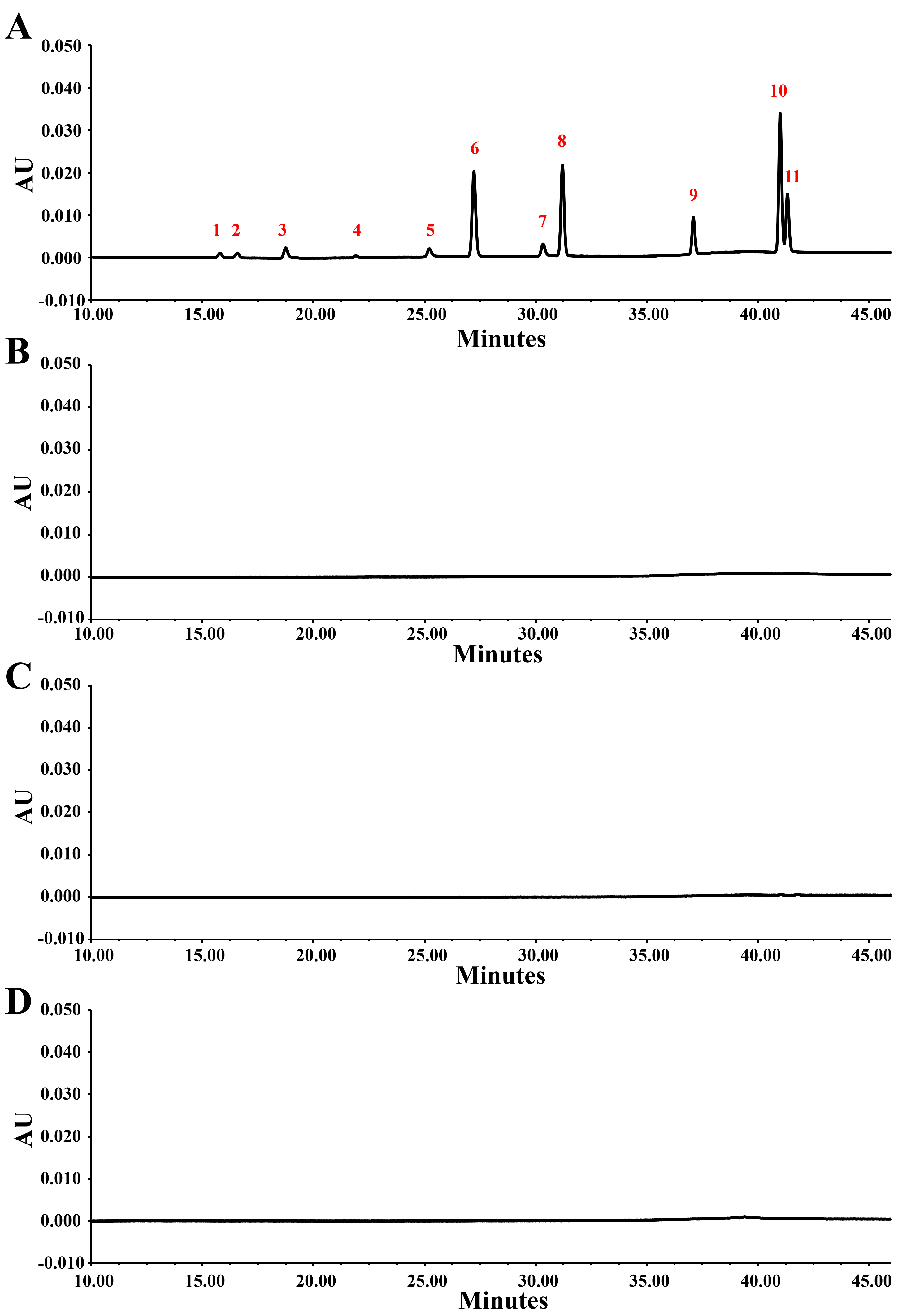


**Figure S2.** HPLC chromatograms of mixed standard solution. HPLC chromatograms of XECQ mixed reference solution (A), methanol (B), 65% ethanol (C), and ultrapure water (D). Note: Peak 1-Salidroside (HJTG), Peak 2-Forsythoside E (LQZGE), Peak 3-Oxypaeoniflorin (YHSYG), Peak 4-Albiflorin (SYNZG), Peak 5-Paeoniflorin (SYG), Peak 6-Daidzin (DDG), Peak 7-Galloylpaeoniflorin (MSZXSYG), Peak 8-Forsythoside A (LQZGA), Peak 9-Ononin (MBHG), Peak 10-Wogonoside (HHQG), Peak 11-Genistein (RLMS).


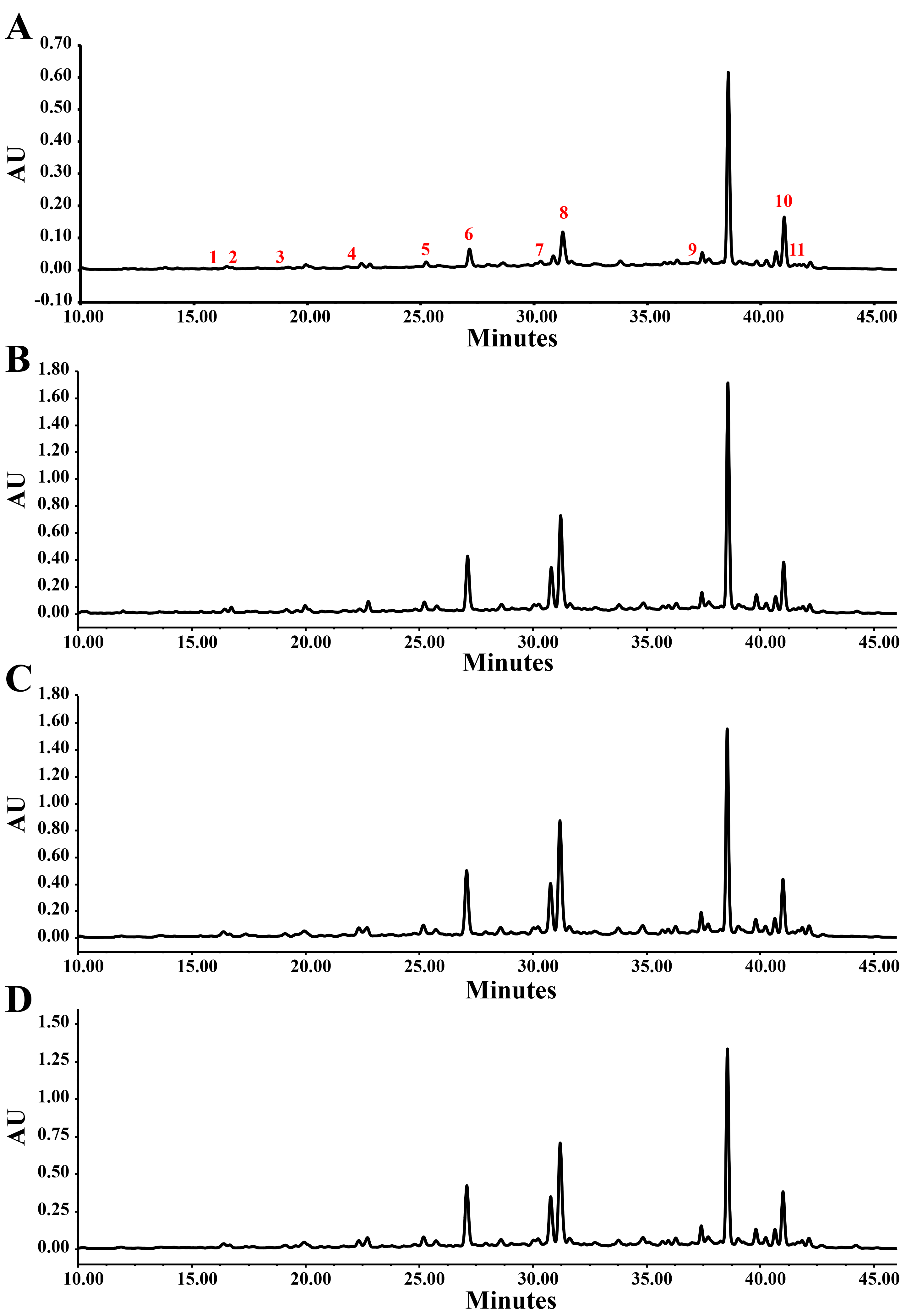


**Figure S3.** HPLC chromatograms of test samples. XECQ water extract (A), Water extract concentrate (B), Alcohol precipitation supernatant (C), Alcohol precipitation concentrates (D). Note: Peak 1-HJTG, Peak 2-LQZGE, Peak 3-YHSYG, Peak 4-SYNZG, Peak 5-SYG, Peak 6-DDG, Peak 7-MSZXSYG, Peak 8-LQZGA, Peak 9-MBHG, Peak 10-HHQG, Peak 11-RLMS.

**Table S1** Calibration curves, regression equations, linear ranges, and correlation coefficients of 11 indicator components.

| Index constituents | Regression equation | Linear range (mg/mL) | Correlation coefficient *R^2^* |
| --- | --- | --- | --- |
| HJTG | y=3.6575x+5.1205 | 0.003663~0.2344 | 0.9997 |
| LQZGE | y=4.1337x+3.6987 | 0.003518~0.2251 | 0.9997 |
| YHSYG | y=9.3926x+2.7903 | 0.004013~0.2568 | 0.9998 |
| SYNZG | y=0.7378x-2.6738 | 0.009139~0.5849 | 0.9994 |
| SYG | y=1.2974x+1.2366 | 0.02435~1.5585 | 0.9998 |
| DDG | y=24.0229x+88.0283 | 0.009320~0.5965 | 0.9997 |
| MSZXSYG | y=11.2638x+10.9240 | 0.003391~0.2170 | 0.9997 |
| LQZGA | y=11.7525x+78.7820 | 0.01856~1.1881 | 0.9997 |
| MBHG | y=22.8614x+30.0819 | 0.003430~0.2195 | 0.9997 |
| HHQG | y=48.4339x+134.5970 | 0.006181~0.3956 | 0.9997 |
| RLMS | y=41.0935x+46.5852 | 0.003262~0.2087 | 0.9997 |

Note: HJTG means salidroside. LQZGE means forsythoside. YHSYG means oxypaeoniflorin. SYNZG means paeonolide. SYG means paeoniflorin. DDG means daidzin. MSZXSYG means galloylpaeoniflorin. LQZGA means forsythoside. MBHG means ononin. HHQG means wogonoside. RLMS means genistein.

**Table S2** Stability test results of XECQ sample.

| Index components | Peak area | | | | | | | | RSD |
| --- | --- | --- | --- | --- | --- | --- | --- | --- | --- |
|  | 0h | 2h | 4h | 6h | 8h | 10h | 12h | 24h |  |
| HJTG | 74816 | 75271 | 76458 | 79999 | 79325 | 79889 | 80117 | 78792 | 2.84 |
| LQZGE | 268944 | 267626 | 264691 | 275385 | 278381 | 282705 | 272844 | 278871 | 2.29 |
| YHSYG | 44634 | 46691 | 47785 | 44871 | 46237 | 46705 | 45675 | 45953 | 2.24 |
| SYNZG | 135204 | 126019 | 137188 | 135073 | 132979 | 134374 | 131877 | 128615 | 2.80 |
| SYG | 1195614 | 1197287 | 1184527 | 1138727 | 1152117 | 1149227 | 1154855 | 1097634 | 2.88 |
| DDG | 5013051 | 5084172 | 4999425 | 5045546 | 5093036 | 5060591 | 5109840 | 4719035 | 2.51 |
| MSZXSYG | 941338 | 957219 | 940290 | 946305 | 959408 | 951284 | 952949 | 917312 | 1.42 |
| LQZGA | 8516941 | 8617814 | 8476182 | 8558027 | 8634382 | 8599154 | 8679787 | 8001733 | 2.53 |
| MBHG | 740368 | 781339 | 748546 | 751311 | 744149 | 745035 | 734078 | 715747 | 2.47 |
| HHQG | 3857027 | 3933757 | 3841157 | 3867647 | 3912894 | 3885461 | 3919886 | 3648762 | 2.35 |
| RLMS | 420583 | 427444 | 421340 | 423995 | 419311 | 406522 | 406539 | 395814 | 2.63 |

**Table S3** Limit of detection (LOD) and quantitation (LOQ) for 11 types of components in XECQ.

| Components | LOD (μg/mL) | LOQ (μg/mL) |
| --- | --- | --- |
| HJTG | 0.6456±0.01 | 2.2098±0.09 |
| LQZGE | 0.6541±0.02 | 1.9146±0.03 |
| YHSYG | 0.6742±0.03 | 0.9302±0.03 |
| SYNZG | 0.7048±0.04 | 11.6619±0.07 |
| SYG | 0.8747±0.09 | 8.2795±0.84 |
| DDG | 0.6242±0.02 | 0.3338±0.00 |
| MSZXSYG | 0.6073±0.00 | 0.6760±0.03 |
| LQZGA | 0.4887±0.04 | 0.5208±0.03 |
| MBHG | 0.5226±0.01 | 0.3223±0.00 |
| HHQG | 0.0301±0.00 | 0.1400±0.00 |
| RLMS | 0.0555±0.01 | 0.1947±0.00 |

**Table S4** Accuracy assessment of selected components based on sample recovery experiments conducted at three spiking levels (n = 3).

| Components | Sample quantity (μg) | Add content (μg) | Measured quantity (μg) | Average recovery rate (%) | RSD (%) |
| --- | --- | --- | --- | --- | --- |
| DDG | 199.43 | 47.719 | 248.784 | 103.24 | 2.31 |
|  |  | 47.719 | 247.238 |  |  |
|  |  | 47.719 | 248.350 |  |  |
|  |  | 59.648 | 262.461 |  |  |
|  |  | 59.648 | 263.299 |  |  |
|  |  | 59.648 | 262.115 |  |  |
|  |  | 71.578 | 271.499 |  |  |
|  |  | 71.578 | 273.516 |  |  |
|  |  | 71.578 | 271.732 |  |  |
| HHQG | 72.49 | 31.648 | 102.895 | 96.79 | 2.94 |
|  |  | 31.648 | 103.658 |  |  |
|  |  | 31.648 | 104.045 |  |  |
|  |  | 39.561 | 111.416 |  |  |
|  |  | 39.561 | 111.107 |  |  |
|  |  | 39.561 | 111.684 |  |  |
|  |  | 47.473 | 115.621 |  |  |
|  |  | 47.473 | 118.593 |  |  |
|  |  | 47.473 | 117.021 |  |  |
| HJTG | 17.88 | 18.754 | 36.607 | 103.36 | 2.96 |
|  |  | 18.754 | 38.183 |  |  |
|  |  | 18.754 | 37.778 |  |  |
|  |  | 23.443 | 41.681 |  |  |
|  |  | 23.443 | 41.428 |  |  |
|  |  | 23.443 | 42.482 |  |  |
|  |  | 28.132 | 46.236 |  |  |
|  |  | 28.132 | 47.773 |  |  |
|  |  | 28.132 | 46.601 |  |  |
| LQZGA | 685.91 | 95.048 | 779.101 | 99.93 | 3.64 |
|  |  | 95.048 | 779.353 |  |  |
|  |  | 95.048 | 782.428 |  |  |
|  |  | 118.810 | 808.015 |  |  |
|  |  | 118.810 | 809.060 |  |  |
|  |  | 118.810 | 810.104 |  |  |
|  |  | 142.572 | 822.059 |  |  |
|  |  | 142.572 | 829.736 |  |  |
|  |  | 142.572 | 820.147 |  |  |
| LQZGE | 58.36 | 18.012 | 75.156 | 97.75 | 2.92 |
|  |  | 18.012 | 76.519 |  |  |
|  |  | 18.012 | 76.401 |  |  |
|  |  | 22.515 | 79.382 |  |  |
|  |  | 22.515 | 80.264 |  |  |
|  |  | 22.515 | 80.389 |  |  |
|  |  | 27.018 | 85.591 |  |  |
|  |  | 27.018 | 85.037 |  |  |
|  |  | 27.018 | 84.670 |  |  |
| MBHG | 29.84 | 17.563 | 47.199 | 102.50 | 2.31 |
|  |  | 17.563 | 47.877 |  |  |
|  |  | 17.563 | 48.104 |  |  |
|  |  | 21.954 | 53.046 |  |  |
|  |  | 21.954 | 51.925 |  |  |
|  |  | 21.954 | 52.696 |  |  |
|  |  | 26.345 | 56.294 |  |  |
|  |  | 26.345 | 57.526 |  |  |
|  |  | 26.345 | 56.505 |  |  |
| MSZXSYG | 81.24 | 17.362 | 99.515 | 103.18 | 2.74 |
|  |  | 17.362 | 99.464 |  |  |
|  |  | 17.362 | 98.783 |  |  |
|  |  | 21.702 | 104.904 |  |  |
|  |  | 21.702 | 103.507 |  |  |
|  |  | 21.702 | 103.571 |  |  |
|  |  | 26.043 | 107.890 |  |  |
|  |  | 26.043 | 107.436 |  |  |
|  |  | 26.043 | 107.293 |  |  |
| RLMS | 8.81 | 16.699 | 25.365 | 97.99 | 2.48 |
|  |  | 16.699 | 25.660 |  |  |
|  |  | 16.699 | 25.770 |  |  |
|  |  | 20.874 | 29.220 |  |  |
|  |  | 20.874 | 29.166 |  |  |
|  |  | 20.874 | 29.405 |  |  |
|  |  | 25.049 | 33.003 |  |  |
|  |  | 25.049 | 32.761 |  |  |
|  |  | 25.049 | 32.351 |  |  |
| SYG | 844.02 | 124.683 | 965.733 | 99.97 | 3.13 |
|  |  | 124.683 | 962.350 |  |  |
|  |  | 124.683 | 967.203 |  |  |
|  |  | 155.854 | 1000.205 |  |  |
|  |  | 155.854 | 1002.484 |  |  |
|  |  | 155.854 | 1009.125 |  |  |
|  |  | 187.025 | 1028.328 |  |  |
|  |  | 187.025 | 1035.231 |  |  |
|  |  | 187.025 | 1030.626 |  |  |
| SYNZG | 165.85 | 46.794 | 212.369 | 103.37 | 2.68 |
|  |  | 46.794 | 214.373 |  |  |
|  |  | 46.794 | 213.866 |  |  |
|  |  | 58.493 | 225.249 |  |  |
|  |  | 58.493 | 225.303 |  |  |
|  |  | 58.493 | 225.732 |  |  |
|  |  | 70.191 | 238.528 |  |  |
|  |  | 70.191 | 241.623 |  |  |
|  |  | 70.191 | 241.286 |  |  |
| YHSYG | 4.27 | 20.547 | 23.860 | 95.10 | 2.22 |
|  |  | 20.547 | 23.964 |  |  |
|  |  | 20.547 | 23.383 |  |  |
|  |  | 25.684 | 28.840 |  |  |
|  |  | 25.684 | 28.121 |  |  |
|  |  | 25.684 | 28.190 |  |  |
|  |  | 30.821 | 33.104 |  |  |
|  |  | 30.821 | 34.401 |  |  |
|  |  | 30.821 | 34.647 |  |  |

**Table S5** Quantitative determination of 11 indicator components in water extracts from different batches.

| Sample | Mass concentration/(μg/mL) | | | | | | | | | | |
| --- | --- | --- | --- | --- | --- | --- | --- | --- | --- | --- | --- |
|  | HJTG | LQZGE | YHSYG | SYNZG | SYG | DDG | MSZXSYG | LQZGA | MBHG | HHQG | RLMS |
| 1-064A | 3.07 | 10.20 | 1.93 | 35.74 | 228.67 | 28.66 | 30.42 | 126.67 | 20.42 | 30.95 | 3.77 |
| 1-074A | 10.31 | 41.31 | 5.65 | 110.91 | 485.96 | 105.08 | 57.26 | 397.63 | 28.41 | 40.60 | 5.73 |
| 1-084A | 13.02 | 24.49 | 4.80 | 136.93 | 332.48 | 36.07 | 60.96 | 181.19 | 45.13 | 37.25 | 5.00 |
| 1-094A | 11.06 | 37.47 | 8.24 | 160.65 | 498.13 | 87.24 | 58.96 | 328.48 | 32.32 | 41.19 | 5.69 |
| 1-104A | 11.51 | 25.03 | 5.93 | 216.99 | 413.72 | 49.87 | 57.71 | 219.91 | 36.67 | 41.20 | 5.35 |
| 1-114A | 9.44 | 20.32 | 4.78 | 157.76 | 375.12 | 37.32 | 58.62 | 184.27 | 40.77 | 36.23 | 4.44 |
| 1-124A | 11.04 | 37.13 | 6.53 | 99.57 | 564.12 | 89.47 | 61.05 | 349.06 | 36.80 | 52.38 | 6.21 |
| 1-134A | 6.93 | 15.21 | 4.20 | 134.26 | 340.69 | 26.29 | 51.14 | 148.61 | 41.43 | 33.62 | 4.01 |
| 1-144A | 10.04 | 40.12 | 7.56 | 125.82 | 487.22 | 79.64 | 50.13 | 295.57 | 31.12 | 35.41 | 4.59 |
| 1-154A | 9.57 | 35.91 | 7.42 | 88.47 | 439.63 | 78.16 | 50.16 | 299.75 | 31.67 | 45.19 | 5.92 |
| 1-164A | 8.91 | 4.17 | 4.74 | 127.09 | 233.88 | 11.23 | 49.18 | 92.64 | 60.48 | 37.31 | 5.47 |
| 1-174A | 6.69 | 34.39 | 6.78 | 103.84 | 467.41 | 80.45 | 50.02 | 304.65 | 28.39 | 58.24 | 6.24 |
| 1-184A | 5.05 | 9.15 | 5.50 | 190.14 | 343.77 | 21.22 | 57.42 | 127.66 | 48.65 | 58.63 | 6.17 |
| 1-194A | 5.66 | 27.99 | 7.35 | 251.42 | 419.09 | 59.77 | 56.31 | 230.71 | 39.77 | 58.85 | 7.30 |
| 1-204A | 5.22 | 30.03 | 7.85 | 204.72 | 485.05 | 67.72 | 61.47 | 281.18 | 39.36 | 63.59 | 8.24 |

**Table S6** Quantitative determination of 11 indicator components in water extract concentrates from different batches.

| Sample | Mass concentration/(μg/mL) | | | | | | | | | | |
| --- | --- | --- | --- | --- | --- | --- | --- | --- | --- | --- | --- |
|  | HJTG | LQZGE | YHSYG | SYNZG | SYG | DDG | MSZXSYG | LQZGA | MBHG | HHQG | RLMS |
| 2-064A | 202.99 | 944.95 | 78.38 | 2464.74 | 8436.56 | 1930.30 | 799.94 | 6916.57 | 397.31 | 743.86 | 97.46 |
| 2-074A | 163.29 | 948.33 | 78.75 | 2735.83 | 7676.6 | 1844.62 | 714.04 | 6417.12 | 343.10 | 936.33 | 93.77 |
| 2-084A | 230.62 | 1110.78 | 80.46 | 2491.16 | 8045.59 | 1979.15 | 883.5 | 6514.08 | 401.30 | 740.94 | 92.34 |
| 2-094A | 207.31 | 959.36 | 84.69 | 2412.03 | 6998.51 | 1954.90 | 792.71 | 7143.55 | 390.18 | 782.65 | 93.71 |
| 2-104A | 208.36 | 951.82 | 90.18 | 2347.05 | 6975.98 | 1873.83 | 768.52 | 6516.08 | 375.90 | 902.1 | 94.71 |
| 2-114A | 111.97 | 760.21 | 92.26 | 1887.74 | 8061.15 | 1775.88 | 771.5 | 6577.6 | 350.28 | 853.81 | 89.8 |
| 2-124A | 115.19 | 880.81 | 81.44 | 1836.07 | 7575.43 | 1857.51 | 771.82 | 6625.6 | 343.41 | 962.71 | 94.26 |
| 2-134A | 165.63 | 965.51 | 81.57 | 1935.35 | 8282.76 | 1886.05 | 761.19 | 6585.58 | 357.93 | 767.98 | 84.77 |
| 2-144A | 299.01 | 1302.59 | 114.09 | 2749.99 | 12039.48 | 2472.05 | 1037.12 | 8812.53 | 511.17 | 1067.09 | 118.15 |
| 2-154A | 203.07 | 918.47 | 88.68 | 2617.67 | 7473.36 | 1761.28 | 793.74 | 6450.84 | 416.38 | 881.67 | 95.95 |
| 2-164A | 91.44 | 718.56 | 74.79 | 1898.87 | 5938.78 | 1378.25 | 696.5 | 4870.65 | 357.75 | 715.81 | 72.37 |
| 2-174A | 95.96 | 702.10 | 81.32 | 2017.63 | 7495.59 | 1485.77 | 743.35 | 5603.29 | 369.08 | 955.04 | 89.71 |
| 2-184A | 95.59 | 705.80 | 92.24 | 2842.11 | 8863.54 | 1439.86 | 816.19 | 5566.83 | 412.43 | 1079.39 | 100.61 |
| 2-194A | 106.84 | 582.14 | 83.09 | 3140.35 | 7148.37 | 1118.61 | 740.55 | 4267.63 | 409.19 | 946.03 | 114.39 |
| 2-204A | 142.66 | 769.37 | 92.12 | 2755.48 | 7490.05 | 1491.03 | 858.26 | 5522.63 | 422.24 | 1124.55 | 131.48 |

**Table S7** Quantitative determination of 11 indicator components in the supernatant of alcohol precipitation from different batches.

| Sample | Mass concentration/(μg/mL) | | | | | | | | | | |
| --- | --- | --- | --- | --- | --- | --- | --- | --- | --- | --- | --- |
|  | HJTG | LQZGE | YHSYG | SYNZG | SYG | DDG | MSZXSYG | LQZGA | MBHG | HHQG | RLMS |
| 3-064A | 41.62 | 108.38 | 20.84 | 520.88 | 2261.65 | 504.33 | 184.66 | 1753.41 | 75.49 | 195.90 | 25.27 |
| 3-074A | 44.66 | 117.89 | 21.65 | 551.17 | 2191.20 | 510.00 | 168.30 | 1726.60 | 68.00 | 211.44 | 20.17 |
| 3-084A | 40.90 | 103.81 | 20.58 | 464.02 | 2185.82 | 474.99 | 173.74 | 1581.92 | 68.02 | 176.81 | 21.01 |
| 3-094A | 40.89 | 107.65 | 20.70 | 354.09 | 1288.41 | 438.34 | 135.75 | 1593.31 | 55.95 | 204.48 | 21.50 |
| 3-104A | 46.84 | 113.29 | 24.94 | 592.87 | 2063.49 | 496.05 | 175.14 | 1665.98 | 71.91 | 236.07 | 24.60 |
| 3-114A | 39.14 | 103.03 | 23.99 | 295.44 | 1787.96 | 486.41 | 168.88 | 1742.89 | 63.03 | 210.54 | 22.99 |
| 3-124A | 39.98 | 111.74 | 26.12 | 446.89 | 2019.99 | 499.37 | 181.84 | 1711.55 | 64.71 | 253.29 | 24.27 |
| 3-134A | 36.51 | 107.50 | 23.01 | 400.27 | 2464.44 | 484.38 | 171.16 | 1639.13 | 66.45 | 198.94 | 21.29 |
| 3-144A | 55.25 | 120.76 | 23.67 | 362.21 | 2117.56 | 516.16 | 144.83 | 1798.18 | 38.49 | 213.13 | 20.19 |
| 3-154A | 51.83 | 113.44 | 20.91 | 318.09 | 1560.68 | 450.37 | 131.96 | 1566.45 | 40.90 | 199.37 | 18.93 |
| 3-164A | 35.83 | 94.35 | 15.69 | 387.84 | 1388.59 | 358.06 | 160.41 | 1247.30 | 67.47 | 169.17 | 17.01 |
| 3-174A | 32.23 | 90.28 | 16.99 | 401.15 | 1802.61 | 385.61 | 173.21 | 1432.39 | 67.73 | 223.78 | 21.40 |
| 3-184A | 30.77 | 76.45 | 16.02 | 569.79 | 1607.11 | 298.24 | 164.71 | 1144.61 | 69.87 | 215.02 | 21.37 |
| 3-194A | 26.39 | 84.66 | 16.33 | 775.52 | 1783.82 | 309.07 | 185.39 | 1131.16 | 84.06 | 229.14 | 27.75 |
| 3-204A | 31.52 | 86.91 | 15.08 | 508.72 | 1316.18 | 336.24 | 173.58 | 1233.46 | 68.19 | 232.22 | 25.84 |

**Table S8** Quantitative determination of 11 indicator components in alcohol precipitation concentrates from different batches.

| Sample | Mass concentration/(μg/mL) | | | | | | | | | | |
| --- | --- | --- | --- | --- | --- | --- | --- | --- | --- | --- | --- |
|  | HJTG | LQZGE | YHSYG | SYNZG | SYG | DDG | MSZXSYG | LQZGA | MBHG | HHQG | RLMS |
| 4-064A | 357.64 | 1167.14 | 85.32 | 3316.94 | 16880.36 | 3988.60 | 1624.86 | 13718.14 | 596.86 | 1449.80 | 176.14 |
| 4-074A | 371.08 | 1198.14 | 130.30 | 3961.20 | 16725.82 | 4172.12 | 1522.34 | 14314.84 | 634.70 | 1753.84 | 190.78 |
| 4-084A | 403.08 | 1206.92 | 127.54 | 3832.98 | 16131.16 | 3998.80 | 1583.28 | 13230.32 | 608.26 | 1375.78 | 164.22 |
| 4-094A | 395.00 | 1112.46 | 133.28 | 4044.24 | 14198.40 | 4026.24 | 1480.70 | 14434.82 | 595.84 | 1516.92 | 171.22 |
| 4-104A | 350.74 | 1057.30 | 150.76 | 4067.32 | 15805.10 | 3789.18 | 1452.76 | 13163.40 | 609.36 | 1745.60 | 176.72 |
| 4-114A | 349.32 | 1073.82 | 156.82 | 3619.80 | 15332.48 | 4061.48 | 1558.10 | 14629.60 | 579.42 | 1675.86 | 169.86 |
| 4-124A | 333.58 | 1085.50 | 128.20 | 2928.46 | 13061.38 | 3855.34 | 1475.64 | 13514.52 | 519.42 | 1704.88 | 161.54 |
| 4-134A | 322.48 | 1077.42 | 132.00 | 3073.06 | 17531.32 | 3844.30 | 1399.44 | 13636.06 | 542.86 | 1418.90 | 144.84 |
| 4-144A | 360.66 | 1110.26 | 149.06 | 3715.40 | 17764.48 | 3937.36 | 1480.74 | 13676.78 | 590.28 | 1344.82 | 149.26 |
| 4-154A | 406.26 | 1133.78 | 156.52 | 3170.10 | 13523.58 | 3695.54 | 1527.48 | 13334.56 | 652.10 | 1694.76 | 174.58 |
| 4-164A | 281.14 | 1059.82 | 135.88 | 3601.60 | 13501.60 | 3375.98 | 1478.66 | 11802.62 | 617.66 | 1658.30 | 138.80 |
| 4-174A | 284.90 | 962.28 | 154.66 | 3879.80 | 16693.76 | 3256.90 | 1450.92 | 12071.72 | 597.06 | 2015.96 | 164.02 |
| 4-184A | 275.00 | 832.18 | 162.10 | 5354.28 | 15481.88 | 2728.22 | 1452.12 | 10616.90 | 623.00 | 1983.94 | 171.40 |
| 4-194A | 216.66 | 767.50 | 133.78 | 5377.42 | 13307.92 | 2418.72 | 1409.60 | 8872.48 | 633.58 | 1827.34 | 205.84 |
| 4-204A | 255.20 | 913.80 | 135.82 | 4636.90 | 12968.52 | 2877.44 | 1480.46 | 10530.14 | 596.74 | 2011.32 | 220.64 |

**Table S9** Plackett-Burman design and responses for terpenoid, flavonoid, phenethyl glycoside retention rates, and solid removal rate.

| Experimental batch | A (%) | B (%) | C (%) | D (mL/min) | E (r/min) | F (^o^C) | G (h) | Y_1_ (%) | Y_2_ (%) | Y_3_ (%) | Y_4_ (%) |
| --- | --- | --- | --- | --- | --- | --- | --- | --- | --- | --- | --- |
| 1 | 20 | 95 | 60 | 60 | 640 | 4 | 24 | 89.93 | 98.38 | 92.20 | 31.80 |
| 2 | 30 | 85 | 60 | 20 | 640 | 4 | 24 | 90.27 | 96.53 | 90.95 | 30.57 |
| 3 | 30 | 95 | 70 | 20 | 640 | 25 | 24 | 80.58 | 85.53 | 83.14 | 36.83 |
| 4 | 30 | 95 | 60 | 20 | 320 | 25 | 12 | 84.87 | 91.70 | 86.20 | 32.08 |
| 5 | 20 | 95 | 70 | 20 | 320 | 4 | 24 | 89.20 | 98.19 | 91.78 | 32.10 |
| 6 | 30 | 85 | 60 | 60 | 320 | 25 | 24 | 89.44 | 97.11 | 90.50 | 34.21 |
| 7 | 20 | 85 | 70 | 20 | 640 | 25 | 12 | 88.37 | 95.76 | 90.47 | 28.84 |
| 8 | 20 | 95 | 60 | 60 | 640 | 25 | 12 | 89.26 | 94.16 | 88.12 | 29.22 |
| 9 | 20 | 85 | 60 | 20 | 320 | 4 | 12 | 92.40 | 98.81 | 92.26 | 28.14 |
| 10 | 30 | 95 | 70 | 60 | 320 | 4 | 12 | 85.01 | 91.58 | 86.64 | 34.42 |
| 11 | 20 | 85 | 70 | 60 | 320 | 25 | 24 | 86.54 | 95.32 | 90.71 | 32.26 |
| 12 | 30 | 85 | 70 | 60 | 640 | 4 | 12 | 87.66 | 94.83 | 89.41 | 32.76 |

Note: A means the solid content of the concentrate. B means the ethanol volume fraction. C means the ethanol content at the endpoint of alcohol precipitation. D means alcohol flow rate. E means stirring speed. F means static temperature. G means standing time. Y_1_ means the retention rates of terpenoid components. Y_2_ means the retention rates of flavonoid components. Y_3_ means the retention rates of phenethyl glycoside components. Y_4_ means solid removal rate.

**Table S10** Box-Behnken experimental design and results for key process units.

| Run | A (%) | B (%) | C (%) | Y_1_ (%) | Y_2_ (%) | Y_3_ (%) | Y_4_ (%) |
| --- | --- | --- | --- | --- | --- | --- | --- |
| 1 | 25 | 85 | 60 | 89.459 | 96.769 | 94.104 | 32.284 |
| 2 | 20 | 95 | 65 | 85.204 | 94.216 | 92.310 | 33.683 |
| 3 | 20 | 90 | 70 | 85.793 | 91.903 | 92.626 | 34.087 |
| 4 | 30 | 85 | 65 | 89.206 | 95.424 | 94.113 | 34.949 |
| 5 | 30 | 90 | 70 | 84.747 | 93.592 | 87.914 | 38.453 |
| 6 | 25 | 95 | 60 | 90.502 | 99.697 | 92.129 | 34.027 |
| 7 | 30 | 95 | 65 | 84.680 | 91.232 | 92.655 | 38.737 |
| 8 | 25 | 90 | 65 | 86.213 | 93.132 | 92.254 | 35.236 |
| 9 | 25 | 90 | 65 | 87.553 | 95.813 | 93.818 | 34.806 |
| 10 | 20 | 85 | 65 | 88.765 | 96.431 | 94.334 | 31.126 |
| 11 | 25 | 90 | 65 | 87.310 | 95.095 | 93.471 | 34.809 |
| 12 | 30 | 90 | 60 | 88.375 | 97.935 | 94.286 | 34.538 |
| 13 | 25 | 95 | 70 | 82.201 | 90.907 | 87.510 | 36.689 |
| 14 | 20 | 90 | 60 | 80.863 | 88.519 | 88.636 | 31.984 |
| 15 | 25 | 85 | 70 | 86.423 | 93.632 | 94.323 | 33.903 |
| 16 | 25 | 90 | 65 | 86.792 | 94.878 | 93.271 | 35.266 |
| 17 | 25 | 90 | 65 | 86.205 | 94.485 | 93.016 | 34.823 |

Note:A means the solid content of the concentrate. B means the ethanol volume fraction. C means the ethanol content at the endpoint of alcohol precipitation. Y_1_ means the retention rates of terpenoid components. Y_2_ means the retention rates of flavonoid components. Y_3_ means the retention rates of phenethyl glycoside components. Y_4_ means solid removal rate.

**Table S11** Digital image texture features of XECQ particles extracted by GLCM analysis.

| File Name | Contrast | Correlation | Energy | Homogeneity |
| --- | --- | --- | --- | --- |
| G01_1.jpg | 208.2019 | 0.8881 | 0.0009 | 0.1809 |
| G01_2.jpg | 223.5600 | 0.8837 | 0.0009 | 0.1773 |
| G01_3.jpg | 188.0248 | 0.8791 | 0.0008 | 0.1864 |
| G01_4.jpg | 216.3611 | 0.8994 | 0.0009 | 0.1822 |
| G01_5.jpg | 237.5464 | 0.8740 | 0.0008 | 0.1725 |
| G02_1.jpg | 194.1181 | 0.8932 | 0.0007 | 0.1844 |
| G02_2.jpg | 191.7190 | 0.8978 | 0.0008 | 0.1848 |
| G02_3.jpg | 190.6136 | 0.9072 | 0.0008 | 0.1880 |
| G02_4.jpg | 216.3012 | 0.8998 | 0.0008 | 0.1797 |
| G02_5.jpg | 181.8635 | 0.9022 | 0.0008 | 0.1899 |
| G03_1.jpg | 217.5406 | 0.8906 | 0.0008 | 0.1796 |
| G03_2.jpg | 196.3121 | 0.8990 | 0.0008 | 0.1860 |
| G03_3.jpg | 180.3950 | 0.8913 | 0.0009 | 0.1896 |
| G03_4.jpg | 203.7776 | 0.8963 | 0.0008 | 0.1842 |
| G03_5.jpg | 185.3045 | 0.8988 | 0.0008 | 0.1895 |
| G04_1.jpg | 187.4169 | 0.9065 | 0.0009 | 0.1898 |
| G04_2.jpg | 178.4448 | 0.9080 | 0.0008 | 0.1907 |
| G04_3.jpg | 213.9090 | 0.8979 | 0.0008 | 0.1832 |
| G04_4.jpg | 211.4301 | 0.8994 | 0.0009 | 0.1826 |
| G04_5.jpg | 201.0025 | 0.9001 | 0.0009 | 0.1868 |
| G05_1.jpg | 205.2539 | 0.8790 | 0.0009 | 0.1815 |
| G05_2.jpg | 222.0018 | 0.8991 | 0.0008 | 0.1785 |
| G05_3.jpg | 231.7405 | 0.8860 | 0.0009 | 0.1766 |
| G05_4.jpg | 225.9047 | 0.8953 | 0.0009 | 0.1772 |
| G05_5.jpg | 189.6971 | 0.9041 | 0.0008 | 0.1891 |
| G06_1.jpg | 206.8467 | 0.8971 | 0.0009 | 0.1819 |
| G06_2.jpg | 186.0472 | 0.9042 | 0.0008 | 0.1882 |
| G06_3.jpg | 192.6421 | 0.9023 | 0.0008 | 0.1854 |
| G06_4.jpg | 211.0770 | 0.9030 | 0.0009 | 0.1827 |
| G06_5.jpg | 201.4942 | 0.8901 | 0.0007 | 0.1834 |
| G07_1.jpg | 159.9979 | 0.9126 | 0.0010 | 0.1977 |
| G07_2.jpg | 200.2544 | 0.9092 | 0.0008 | 0.1834 |
| G07_3.jpg | 201.7371 | 0.8993 | 0.0008 | 0.1824 |
| G07_4.jpg | 172.9942 | 0.8998 | 0.0009 | 0.1913 |
| G07_5.jpg | 195.4483 | 0.8922 | 0.0007 | 0.1842 |
| G08_1.jpg | 212.9781 | 0.8920 | 0.0008 | 0.1803 |
| G08_2.jpg | 168.6003 | 0.9032 | 0.0008 | 0.1924 |
| G08_3.jpg | 193.8470 | 0.9110 | 0.0008 | 0.1873 |
| G08_4.jpg | 173.9456 | 0.9050 | 0.0008 | 0.1943 |
| G08_5.jpg | 206.7243 | 0.8989 | 0.0008 | 0.1832 |
| G09_1.jpg | 223.2718 | 0.8795 | 0.0007 | 0.1746 |
| G09_2.jpg | 222.6612 | 0.8913 | 0.0008 | 0.1756 |
| G09_3.jpg | 224.0664 | 0.8893 | 0.0009 | 0.1813 |
| G09_4.jpg | 210.7777 | 0.8767 | 0.0008 | 0.1768 |
| G09_5.jpg | 220.2820 | 0.9011 | 0.0008 | 0.1811 |
| G10_1.jpg | 231.6223 | 0.8824 | 0.0007 | 0.1737 |
| G10_2.jpg | 213.6918 | 0.9007 | 0.0007 | 0.1779 |
| G10_3.jpg | 192.5330 | 0.8942 | 0.0008 | 0.1843 |
| G10_4.jpg | 195.2742 | 0.9014 | 0.0007 | 0.1853 |
| G10_5.jpg | 190.5052 | 0.8864 | 0.0009 | 0.1857 |
| G11_1.jpg | 188.3879 | 0.8941 | 0.0009 | 0.1871 |
| G11_2.jpg | 190.4170 | 0.9051 | 0.0008 | 0.1878 |
| G11_3.jpg | 141.1470 | 0.8945 | 0.0008 | 0.2040 |
| G11_4.jpg | 188.8375 | 0.8986 | 0.0010 | 0.1914 |
| G11_5.jpg | 219.5722 | 0.8846 | 0.0008 | 0.1765 |
| G12_1.jpg | 187.5888 | 0.9078 | 0.0009 | 0.1910 |
| G12_2.jpg | 221.1230 | 0.8857 | 0.0009 | 0.1775 |
| G12_3.jpg | 186.5707 | 0.9029 | 0.0009 | 0.1904 |
| G12_4.jpg | 166.2617 | 0.9083 | 0.0009 | 0.1955 |
| G12_5.jpg | 229.3129 | 0.8870 | 0.0008 | 0.1766 |
| G13_1.jpg | 226.7882 | 0.8785 | 0.0008 | 0.1737 |
| G13_2.jpg | 204.9943 | 0.8987 | 0.0008 | 0.1818 |
| G13_3.jpg | 195.7618 | 0.8948 | 0.0009 | 0.1872 |
| G13_4.jpg | 211.0590 | 0.8876 | 0.0008 | 0.1795 |
| G13_5.jpg | 121.7273 | 0.9131 | 0.0009 | 0.2151 |
| G14_1.jpg | 258.3992 | 0.8951 | 0.0007 | 0.1721 |
| G14_2.jpg | 199.8997 | 0.9031 | 0.0008 | 0.1836 |
| G14_3.jpg | 208.0429 | 0.9081 | 0.0007 | 0.1843 |
| G14_4.jpg | 223.0398 | 0.8967 | 0.0007 | 0.1775 |
| G14_5.jpg | 199.1949 | 0.9083 | 0.0007 | 0.1873 |
| RG1_1.jpg | 215.2459 | 0.9379 | 0.0007 | 0.2154 |
| RG1_2.jpg | 162.1913 | 0.9526 | 0.0007 | 0.2336 |
| RG1_3.jpg | 159.5390 | 0.9600 | 0.0007 | 0.2387 |
| RG1_4.jpg | 187.8221 | 0.9440 | 0.0007 | 0.2237 |
| RG1_5.jpg | 153.2841 | 0.9652 | 0.0008 | 0.2443 |
| RG2_1.jpg | 197.8967 | 0.9442 | 0.0004 | 0.1997 |
| RG2_2.jpg | 165.6367 | 0.9504 | 0.0005 | 0.2147 |
| RG2_3.jpg | 178.7151 | 0.9438 | 0.0005 | 0.2057 |
| RG2_4.jpg | 161.9939 | 0.9546 | 0.0005 | 0.2180 |
| RG2_5.jpg | 189.6860 | 0.9390 | 0.0005 | 0.2005 |
| RG3_1.jpg | 145.2736 | 0.9587 | 0.0005 | 0.2254 |
| RG3_2.jpg | 124.9105 | 0.9676 | 0.0005 | 0.2343 |
| RG3_3.jpg | 131.2724 | 0.9651 | 0.0006 | 0.2318 |
| RG3_4.jpg | 129.5173 | 0.9631 | 0.0006 | 0.2365 |
| RG3_5.jpg | 105.8791 | 0.9692 | 0.0006 | 0.2488 |
| RG4_1.jpg | 248.6926 | 0.9304 | 0.0006 | 0.1834 |
| RG4_2.jpg | 181.7347 | 0.9326 | 0.0006 | 0.2005 |
| RG4_3.jpg | 204.7529 | 0.9410 | 0.0006 | 0.1960 |
| RG4_4.jpg | 194.2233 | 0.9487 | 0.0006 | 0.2012 |
| RG4_5.jpg | 241.1682 | 0.9157 | 0.0006 | 0.1805 |
| RG5_1.jpg | 155.0016 | 0.9546 | 0.0007 | 0.2202 |
| RG5_2.jpg | 161.9446 | 0.9543 | 0.0007 | 0.2163 |
| RG5_3.jpg | 156.0595 | 0.9575 | 0.0007 | 0.2172 |
| RG5_4.jpg | 151.9364 | 0.9587 | 0.0006 | 0.2224 |
| RG5_5.jpg | 106.7418 | 0.9481 | 0.0006 | 0.2342 |

**Table S12** Spatial distribution characteristics of standard and reference XECQ granules.

| File Name | Density | Num Particles | Mean Area | Std Area |
| --- | --- | --- | --- | --- |
| G01_1.jpg | 0.8034 | 2744 | 42.2613 | 48.9854 |
| G01_2.jpg | 0.8053 | 2801 | 41.2995 | 46.6779 |
| G01_3.jpg | 0.7864 | 2741 | 42.1295 | 46.9740 |
| G01_4.jpg | 0.8011 | 2481 | 47.6522 | 56.0478 |
| G01_5.jpg | 0.7889 | 2828 | 40.5428 | 47.6721 |
| G02_1.jpg | 0.7658 | 2433 | 48.3662 | 57.0891 |
| G02_2.jpg | 0.7724 | 2536 | 46.1479 | 55.2831 |
| G02_3.jpg | 0.7631 | 2269 | 52.3870 | 62.7365 |
| G02_4.jpg | 0.7655 | 2293 | 51.7588 | 63.5015 |
| G02_5.jpg | 0.7770 | 2424 | 48.7496 | 55.9956 |
| G03_1.jpg | 0.7722 | 2421 | 48.5960 | 60.7708 |
| G03_2.jpg | 0.7713 | 2405 | 48.9530 | 60.3304 |
| G03_3.jpg | 0.7879 | 2628 | 44.4669 | 54.3321 |
| G03_4.jpg | 0.7748 | 2350 | 50.3757 | 63.9002 |
| G03_5.jpg | 0.7776 | 2248 | 53.0343 | 68.1176 |
| G04_1.jpg | 0.7841 | 2299 | 51.9356 | 66.9141 |
| G04_2.jpg | 0.7717 | 2291 | 52.0009 | 67.9664 |
| G04_3.jpg | 0.7723 | 2320 | 51.0720 | 62.9755 |
| G04_4.jpg | 0.7884 | 2390 | 49.5657 | 59.1319 |
| G04_5.jpg | 0.7826 | 2278 | 52.3889 | 68.4271 |
| G05_1.jpg | 0.7971 | 2829 | 40.7925 | 49.2914 |
| G05_2.jpg | 0.7700 | 2377 | 49.5435 | 61.7371 |
| G05_3.jpg | 0.7935 | 2686 | 43.2569 | 49.9401 |
| G05_4.jpg | 0.7913 | 2628 | 44.5537 | 53.5207 |
| G05_5.jpg | 0.7648 | 2137 | 56.0861 | 70.7531 |
| G06_1.jpg | 0.7873 | 2536 | 46.1313 | 52.0384 |
| G06_2.jpg | 0.7740 | 2392 | 49.3825 | 57.9976 |
| G06_3.jpg | 0.7679 | 2454 | 47.9372 | 59.0466 |
| G06_4.jpg | 0.7783 | 2198 | 54.4349 | 62.7438 |
| G06_5.jpg | 0.7645 | 2484 | 47.2786 | 57.0104 |
| G07_1.jpg | 0.8028 | 2384 | 49.8129 | 59.7152 |
| G07_2.jpg | 0.7703 | 2279 | 51.9996 | 62.5090 |
| G07_3.jpg | 0.7658 | 2435 | 48.1918 | 58.8774 |
| G07_4.jpg | 0.7955 | 2442 | 48.4292 | 57.0521 |
| G07_5.jpg | 0.7706 | 2503 | 46.6021 | 54.8318 |
| G08_1.jpg | 0.7711 | 2500 | 46.7948 | 58.0585 |
| G08_2.jpg | 0.7690 | 2369 | 49.9358 | 65.0647 |
| G08_3.jpg | 0.7542 | 2200 | 54.1595 | 73.3807 |
| G08_4.jpg | 0.7710 | 2150 | 55.8805 | 77.6484 |
| G08_5.jpg | 0.7746 | 2359 | 50.1619 | 61.4218 |
| G09_1.jpg | 0.7721 | 2662 | 43.4369 | 50.4192 |
| G09_2.jpg | 0.7708 | 2519 | 46.3152 | 59.6607 |
| G09_3.jpg | 0.7798 | 2589 | 44.9258 | 51.6106 |
| G09_4.jpg | 0.7826 | 2722 | 42.4151 | 49.8668 |
| G09_5.jpg | 0.7701 | 2242 | 53.1574 | 70.6911 |
| G10_1.jpg | 0.7677 | 2716 | 42.4190 | 52.1291 |
| G10_2.jpg | 0.7609 | 2475 | 47.2679 | 56.5249 |
| G10_3.jpg | 0.7753 | 2607 | 44.6344 | 53.7864 |
| G10_4.jpg | 0.7570 | 2316 | 51.1347 | 62.5795 |
| G10_5.jpg | 0.7943 | 2668 | 43.6087 | 52.2105 |
| G11_1.jpg | 0.7944 | 2596 | 45.0732 | 55.4808 |
| G11_2.jpg | 0.7767 | 2377 | 49.9369 | 66.5720 |
| G11_3.jpg | 0.7771 | 2577 | 45.2561 | 54.1664 |
| G11_4.jpg | 0.7963 | 2383 | 49.8703 | 60.6770 |
| G11_5.jpg | 0.7853 | 2642 | 44.0799 | 53.1032 |
| G12_1.jpg | 0.7796 | 2255 | 52.8639 | 62.8821 |
| G12_2.jpg | 0.7879 | 2745 | 42.1373 | 48.1664 |
| G12_3.jpg | 0.7699 | 2334 | 50.8792 | 63.9419 |
| G12_4.jpg | 0.7806 | 2230 | 53.6256 | 63.1247 |
| G12_5.jpg | 0.7828 | 2529 | 46.3685 | 54.7817 |
| G13_1.jpg | 0.7737 | 2767 | 41.5070 | 49.6679 |
| G13_2.jpg | 0.7799 | 2621 | 44.5654 | 55.7893 |
| G13_3.jpg | 0.7843 | 2565 | 45.5591 | 54.3499 |
| G13_4.jpg | 0.7745 | 2585 | 45.0495 | 55.5851 |
| G13_5.jpg | 0.7757 | 2153 | 55.6930 | 65.2726 |
| G14_1.jpg | 0.7564 | 2361 | 49.6929 | 61.1016 |
| G14_2.jpg | 0.7625 | 2272 | 52.1743 | 62.7998 |
| G14_3.jpg | 0.7528 | 2170 | 54.8442 | 65.4557 |
| G14_4.jpg | 0.7602 | 2192 | 54.2536 | 65.8488 |
| G14_5.jpg | 0.7537 | 2004 | 60.2715 | 71.6557 |
| RG1_1.jpg | 0.7676 | 1079 | 120.7312 | 198.0639 |
| RG1_2.jpg | 0.7651 | 999 | 132.0400 | 219.9156 |
| RG1_3.jpg | 0.7632 | 815 | 164.7423 | 257.1564 |
| RG1_4.jpg | 0.7664 | 1048 | 125.0267 | 194.1974 |
| RG1_5.jpg | 0.7620 | 758 | 178.9446 | 286.1464 |
| RG2_1.jpg | 0.7309 | 979 | 134.0817 | 245.7402 |
| RG2_2.jpg | 0.7463 | 1005 | 130.7015 | 291.5166 |
| RG2_3.jpg | 0.7500 | 1140 | 113.5956 | 159.2858 |
| RG2_4.jpg | 0.7397 | 853 | 156.8441 | 264.9976 |
| RG2_5.jpg | 0.7558 | 1242 | 103.6393 | 166.2461 |
| RG3_1.jpg | 0.7221 | 792 | 169.5253 | 623.6376 |
| RG3_2.jpg | 0.7325 | 725 | 187.4690 | 422.9657 |
| RG3_3.jpg | 0.7289 | 757 | 178.5575 | 347.4526 |
| RG3_4.jpg | 0.7322 | 762 | 176.9711 | 362.1994 |
| RG3_5.jpg | 0.7340 | 696 | 195.0876 | 348.7712 |
| RG4_1.jpg | 0.7391 | 1447 | 87.1735 | 131.3862 |
| RG4_2.jpg | 0.7438 | 1276 | 100.2006 | 165.8159 |
| RG4_3.jpg | 0.7363 | 1141 | 113.8449 | 169.4065 |
| RG4_4.jpg | 0.7326 | 944 | 139.8676 | 273.1528 |
| RG4_5.jpg | 0.7645 | 1767 | 69.8478 | 96.2398 |
| RG5_1.jpg | 0.7235 | 723 | 186.7012 | 396.9876 |
| RG5_2.jpg | 0.7331 | 791 | 169.7535 | 301.3188 |
| RG5_3.jpg | 0.7209 | 825 | 162.4824 | 329.7760 |
| RG5_4.jpg | 0.7217 | 834 | 160.9101 | 280.7724 |
| RG5_5.jpg | 0.7275 | 1063 | 122.4788 | 259.7701 |

**Table S13** Particle quality assessment results of XECQ granules based on multi-dimensional feature fusion.

| File name | Total score | Mahalanobis | Color hist score | Color stats score | Texture score | Density score | Decision | Reason |
| --- | --- | --- | --- | --- | --- | --- | --- | --- |
| G12_1.jpg | 96.6 | 0.995 | 89.8 | 93.4 | 100 | 100 | Accept | moderate_score |
| G12_2.jpg | 98.6 | 0.998 | 93.1 | 100.0 | 100 | 100 | Accept |  |
| G12_3.jpg | 94.7 | 0.990 | 88.0 | 85.4 | 100 | 100 | Review |  |
| G12_4.jpg | 96.9 | 0.996 | 90.3 | 94.3 | 100 | 100 | Accept |  |
| G12_5.jpg | 98.2 | 0.997 | 91.0 | 100.0 | 100 | 100 | Accept |  |
| G13_1.jpg | 98.8 | 0.999 | 94.1 | 100.0 | 100 | 100 | Accept |  |
| G13_2.jpg | 98.8 | 0.999 | 94.1 | 100.0 | 100 | 100 | Accept |  |
| G13_3.jpg | 98.8 | 0.998 | 93.8 | 100.0 | 100 | 100 | Accept |  |
| G13_4.jpg | 98.8 | 0.999 | 94.0 | 100.0 | 100 | 100 | Accept |  |
| G13_5.jpg | 88.6 | 0.994 | 92.1 | 100.0 | 67.1 | 100 | Review |  |
| G14_1.jpg | 98.6 | 0.998 | 93.6 | 100.0 | 99.7 | 100 | Accept |  |
| G14_2.jpg | 98.7 | 0.999 | 93.5 | 100.0 | 100 | 100 | Accept |  |
| G14_3.jpg | 98.5 | 0.997 | 92.4 | 100.0 | 100 | 100 | Accept |  |
| G14_4.jpg | 98.5 | 0.998 | 92.6 | 100.0 | 100 | 100 | Accept |  |
| G14_5.jpg | 98.0 | 0.995 | 92.5 | 100.0 | 100 | 98.2 | Accept |  |
| RG1_1.jpg | 46.6 | 0.089 | 77.5 | 21.8 | 63.4 | 25.9 | Reject | abnormal_dist+low_score |
| RG1_2.jpg | 40.8 | 0.046 | 76.1 | 9.5 | 53.2 | 25.7 | Reject | abnormal_dist+low_score |
| RG1_3.jpg | 40.6 | 0.003 | 73.5 | 14.0 | 51.8 | 25.3 | Reject | abnormal_dist+low_score |
| RG1_4.jpg | 42.2 | 0.058 | 75.8 | 11.3 | 57 | 25.8 | Reject | abnormal_dist+low_score |
| RG1_5.jpg | 40.4 | 0.001 | 72.7 | 15.0 | 51.1 | 25.3 | Reject | abnormal_dist+low_score |
| RG2_1.jpg | 44.8 | 0.124 | 86.6 | 28.9 | 57.6 | 14.9 | Reject | abnormal_dist+low_score |
| RG2_2.jpg | 41.9 | 0.150 | 86.2 | 26.4 | 38.9 | 25.7 | Reject | abnormal_dist+low_score |
| RG2_3.jpg | 46.9 | 0.304 | 86.6 | 29.8 | 52.6 | 26.1 | Reject | abnormal_dist+low_score |
| RG2_4.jpg | 39.9 | 0.019 | 82.3 | 28.1 | 35.6 | 23.8 | Reject | abnormal_dist+low_score |
| RG2_5.jpg | 50.5 | 0.490 | 88.6 | 30.1 | 62.5 | 26.7 | Reject | abnormal_dist+low_score |
| RG3_1.jpg | 38.0 | 0.006 | 90.4 | 34.6 | 34.4 | 9.0 | Reject | abnormal_dist+low_score |
| RG3_2.jpg | 34.7 | 0.001 | 89.3 | 28.4 | 21.3 | 15.8 | Reject | abnormal_dist+low_score |
| RG3_3.jpg | 37.2 | 0.004 | 89.5 | 31.7 | 30.1 | 13.0 | Reject | abnormal_dist+low_score |
| RG3_4.jpg | 37.2 | 0.005 | 89.3 | 30.2 | 28.9 | 15.6 | Reject | abnormal_dist+low_score |
| RG3_5.jpg | 38.9 | 0.001 | 90.3 | 37.2 | 27.4 | 17.2 | Reject | abnormal_dist+low_score |
| RG4_1.jpg | 67.2 | 0.820 | 97.6 | 75.7 | 81.7 | 26.7 | Reject | abnormal_dist+low_score |
| RG4_2.jpg | 67.4 | 0.653 | 97.7 | 75.8 | 82.2 | 27.0 | Reject | abnormal_dist+low_score |
| RG4_3.jpg | 62.4 | 0.410 | 96.4 | 75.6 | 73.0 | 20.4 | Reject | abnormal_dist+low_score |
| RG4_4.jpg | 61.4 | 0.119 | 94.1 | 74.3 | 76.1 | 16.2 | Reject | abnormal_dist+low_score |
| RG4_5.jpg | 81.5 | 0.968 | 99.3 | 92.0 | 96.8 | 47.2 | Reject | low_score |
| RG5_1.jpg | 44.4 | 0.002 | 89.9 | 34.7 | 55.3 | 9.6 | Reject | abnormal_dist+low_score |
| RG5_2.jpg | 47.3 | 0.011 | 90.2 | 35.9 | 57.2 | 16.5 | Reject | abnormal_dist+low_score |
| RG5_3.jpg | 43.2 | 0.025 | 87.3 | 31.8 | 56.2 | 8.5 | Reject | abnormal_dist+low_score |
| RG5_4.jpg | 42.7 | 0.023 | 88.3 | 30.7 | 54.0 | 8.9 | Reject | abnormal_dist+low_score |
| RG5_5.jpg | 42.5 | 0.330 | 91.0 | 48.8 | 35.7 | 12.6 | Reject | abnormal_dist+low_score |


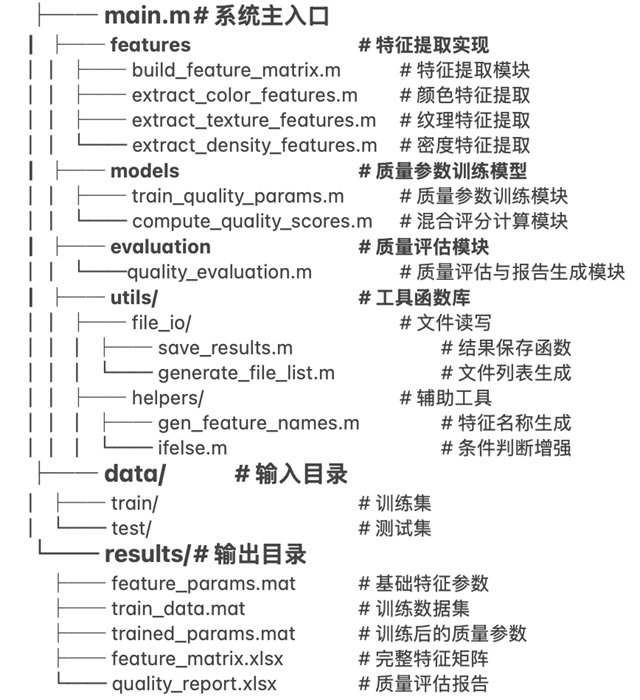


**Figure S4.** Structural framework of the particle quality assessment system
